# Nanocarrier Systems for Plant-Derived Bioactives: Design, Biosafety, and Translational Challenges

**DOI:** 10.3390/pharmaceutics18091148

**Published:** 2026-09-11

**Authors:** Saima Jan, Gulam Rabbani, Arif Tasleem Jan, Khurshid Ahmad

**Affiliations:** 1 Department of Botany, School of Biosciences and Biotechnology, Baba Ghulam Shah University, Rajouri 185234, India; 2 School of Chemical Engineering, Yeungnam University, Gyeongsan 38541, Republic of Korea; 3 Department of Health Informatics, College of Applied Medical Sciences, Qassim University, Buraydah 51452, Saudi Arabia

**Keywords:** plants, secondary metabolites, drug delivery, phyto-therapeutics, disease treatment, phytopharmacology

## Abstract

Plant-derived bioactives offer diverse pharmacological activities, but their development is often limited by poor aqueous solubility, instability, rapid metabolism, and low tissue exposure. This review examines delivery platforms used to address these constraints, including liposomes, solid lipid nanoparticles, nanostructured lipid carriers, phytosomes, niosomes, polymeric and protein-based carriers, extracellular vesicles, and self-assembled polyphenol systems. The platforms are compared according to payload compatibility, loading and release behavior, biological barriers, manufacturing requirements, and formulation-specific safety risks. Though carriers of natural origin offer a sustainable and often less toxic alternative, their ability to induce immunogenicity, organ accumulation, and repeated-dose toxicity require product-specific assessment. The review also discusses sustainable extraction and formulation, emphasizing that green performance depends on the entire process rather than on the feedstock alone. Preclinical studies frequently report improved stability, exposure, or therapeutic activity, whereas human evidence remains limited and is concentrated in early-phase studies of curcumin and silybin formulations and a small number of plant-derived extracellular-vesicle preparations. Translation will require standardized characterization, defined critical quality attributes, scalable Good Manufacturing Practice-compliant production, batch consistency, comparative pharmacokinetic and toxicological studies, and appropriately powered clinical trials. These considerations support rational carrier selection based on the physicochemical properties of the bioactive, intended route and target, release requirements, and strength of the available evidence.

## 1. Introduction

Humans have acknowledged and harnessed the healing properties of medicinal plants since ancient times. Representing a valuable contribution from nature, medicinal plants have yielded a plethora of bioactive compounds in different amounts that serve the purpose of use against various diseases [1]. Considering the utilization of medicinal plants for therapeutic purposes, the World Health Organization (WHO) reported that almost 80% of individuals in both developed and developing countries rely on plant-based remedies in primary healthcare [2]. Bioactive compounds, such as alkaloids, glycosides, tannins, volatile oils, and terpenoids, characterized by their extensive structural complexity and diversity, play a crucial role in addressing various physiological and pathological conditions, including infectious diseases and other disorders [3]. Although medicinal plants and their constituents are widely used, and many demonstrate pharmacological activity in experimental models, their efficacy and safety vary with chemical composition, dose, formulation, route of administration, and quality control. Traditional use or natural origin alone does not establish clinical effectiveness or safety [4,5].

Bioactive compounds, including alkaloids, steroids, terpenoids, polyunsaturated fatty acids, and others, have demonstrated notable efficacy in the treatment of various diseases [6]. However, the challenges inherent in delivering them to target sites greatly influence their stability, efficacy, and, as such, therapeutic effectiveness. In this regard, the application of a suitable delivery system not only enhances their bioavailability but also extends their pharmacological activity by facilitating safe passage across cellular membranes to target cells. The delivery systems are strategically engineered to regulate the release rate of bioactive compounds, their absorption and distribution, and finally their elimination from the body [7]. As delivery systems prove instrumental in transporting small compounds effectively, current research is underway to develop a broad range of lipid-based nanoparticles, such as liposomes, polymeric nanoparticles, and exosomes, as well as composite molecules, for the effective delivery of natural-origin molecules. The synergistic combination of drug-delivery systems with natural products has demonstrated therapeutic benefits in diseases such as diabetes [8], cancer, and neurodegenerative diseases [9]. The present review evaluates how these distinct carrier platforms are used to improve stability, bioavailability, controlled release, and tissue delivery of plant-derived bioactives. Particular emphasis is placed on comparative design principles, biosafety, manufacturing and regulatory constraints, and the current level of clinical evidence.

## 2. Plant-Based Bioactive Compounds

**Figure 1:**
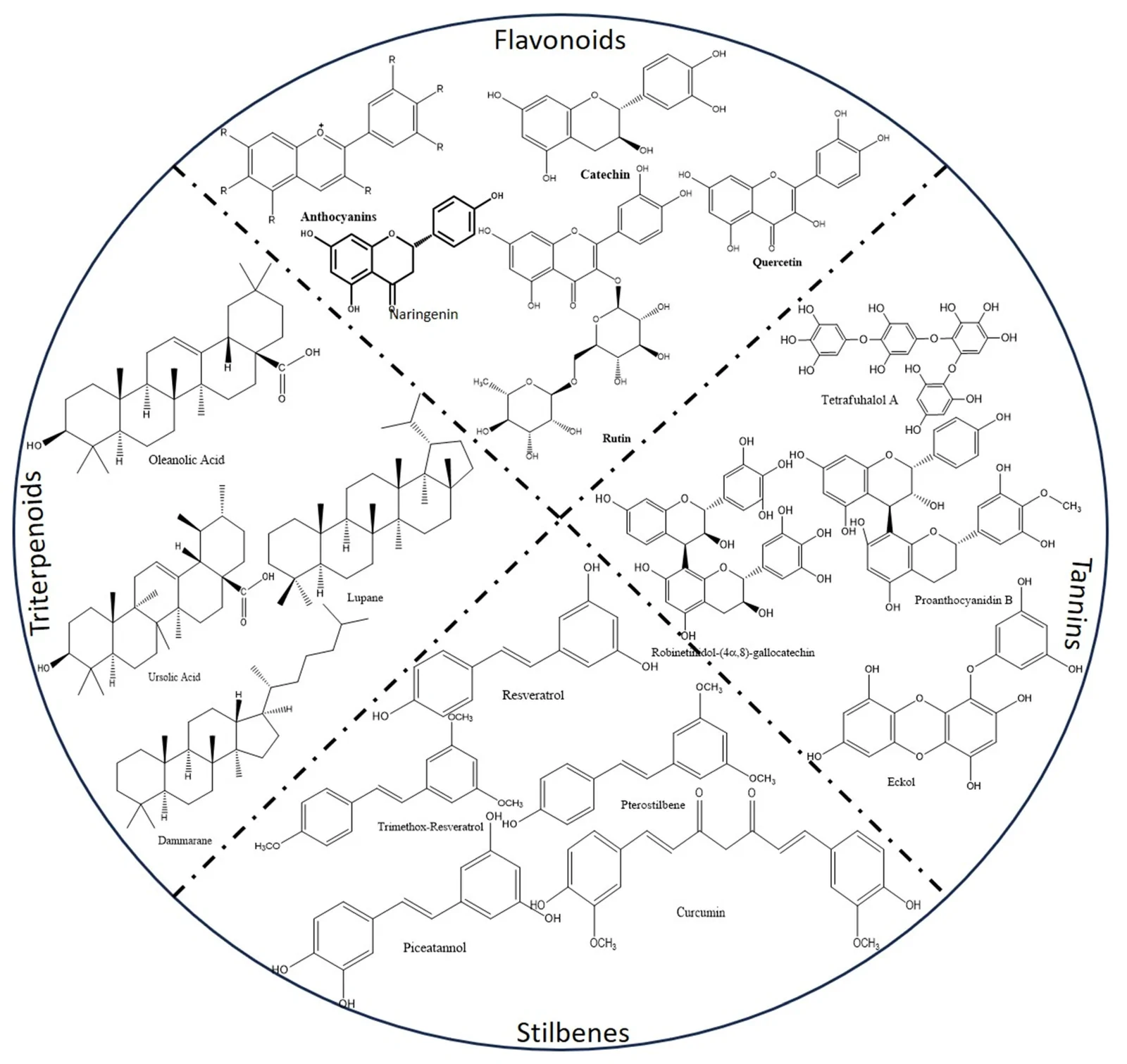
Representative low-molecular-weight plant specialized metabolites discussed in this review.

Plants produce diverse range of primary and specialized metabolites that contribute to adaptation to biotic and abiotic stresses. Many of these constituents also exhibit biological activities relevant to food, pharmaceutical, nutraceutical, and cosmetic applications [10,11,12]. Major classes of plant specialized metabolites include phenolic compounds, such as flavonoids, phenolic acids, tannins, and stilbenes; terpenoids; alkaloids; and other nitrogen-containing compounds. Natural macromolecules, including polysaccharides, proteins, and peptides, should be treated separately because they are not subclasses of low-molecular-weight secondary metabolites (Figure 1).

### 2.1. Flavonoids

Flavonoids constitute a distinct and prevalent subgroup of polyphenols. They include a broad range of bioactive compounds under the sub-head of flavanols, flavanones, isoflavones, flavones, flavonols, proanthocyanidins, and anthocyanins [13]. With fruits such as blueberries, raspberries, and cranberries as the main sources, more than 8000 distinct flavonoids, which attribute distinct colors to fruits, flowers, etc., have been recognized [14]. Common flavonoids, including rutin, kaempferol, and quercetin, are known for their robust antioxidant properties [15]. Rutin, an abundant flavonoid, constitutes a major proportion (90%) of the total phenolics [16]. Flavonols such as kaempferol and quercetin have been successfully identified and isolated [17,18,19]. Several isoflavones, including genistin, glycitin, and others, have been isolated and characterized [17]. Also, four proanthocyanidins, namely procyanidin A2, -A3, B2, and B3, along with two anthocyanins, specifically cyanidin 3-O-glucoside and cyanidin 3-O-rutinoside, have also been identified among different plant species [17,18]. Additionally, fagopyrin A–F have been identified in the realm of phytochemical analysis of Fagopyrum species [20].

*Quercetin*, a prevalent polyphenolic compound found in raspberries, cherries, apples, grapes, blueberries, and blackcurrants [21], is recognized as an exceptionally effective scavenger of reactive oxygen species (ROS) within the flavonoid category, specifically belonging to the flavonol subclass [22]. A proposed experimental investigation aims to assess the capacity of flavonoids to scavenge free radicals in human myelogenous leukemia (K562) cells subjected to hydrogen peroxide (H_2_O_2_) treatment [23]. Chondrogianni et al. [24] demonstrated that quercetin and its derivative, quercetin caprylate (QU-CAP), can revitalize senescent human fibroblasts, extending their lifespan by activating the proteasome. Quercetin inhibits the generation of inflammatory compounds, including lipoxygenase (LOX) and cyclooxygenase (COX) products, thereby reducing the synthesis of prostaglandins and leukotrienes, substances known to promote inflammation [25].

*Anthocyanins* form a unique spectrum of colors commonly present in nature, occupying a significant position within the polyphenol group [26]. These compounds are extensively distributed in a diverse range of foods, with elevated concentrations typically found in bilberries, raspberries, blackberries, cranberries, blackcurrants, strawberries, and blueberries [27]. Despite the identification of over 600 anthocyanins in nature, only six—peonidin, delphinidin, malvidin, pelargonidin, cyanidin, and petunidin—are commonly found in fruits [28]. The antioxidant properties of anthocyanins are linked to the existence of free hydroxyl groups surrounding the pyrrole ring. The higher the count of hydroxyl groups, the greater the associated antioxidant activity [29]. Anthocyanins have been shown to suppress free radical formation, thereby reducing the incidence of many age-related conditions such as cardiovascular diseases, delaying the aging process, and enhancing memory [30]. Anthocyanins are predominantly located in the outer layer and are situated in the pericarp of fruits. They exhibit a wide range of properties, including antioxidant, anticancer, and neuroprotective effects [31]. Cyanidin-3-glucoside stands out as the primary anthocyanin present in the majority of fruits.

*Catechin*, found in berries such as gooseberries, cherries, blueberries, etc., and apples, includes gallocatechin, epicatechin (EC), epicatechin 3-gallate (ECG), epigallocatechin 3-gallate (EGCG), and epigallocatechin (EGC). It is estimated that apples contain catechins in the range of 10–43 mg/100 g, with a notable abundance of EGCG, while strawberries and cherries contain 2–50 mg/100 g and 5–22 mg/100 g, respectively [32]. Catechins are primarily located in the outer tissues of fruit, containing ketone groups, particularly rutin and quercetin, which have antioxidant properties. In cranberries, the catechin present is similar to that in green tea, offering protective benefits against cancer [33]. The potent antioxidant capabilities of catechin extend to combating cancer, neurodegenerative conditions, and cardiovascular diseases [34]. Epigallocatechin gallate (EGCG), a significant catechin, inhibits telomerase, a crucial enzyme responsible for extending the lifespan of diseased cells by maintaining their chromosome ends. This action could serve as another explanation for the anticancer properties of catechins. Catechins play a role in preventing coronary heart disease by reducing cytotoxicity induced by amiodarone in lung fibroblast cells [35]. Additionally, catechins contribute to lessening neuronal degeneration via a reduction in the levels of reactive oxygen species (ROS), and a subsequent enhancement in the production of antioxidant enzymes [35].

The number and locations of the hydroxyl (-OH) groups in the structure of flavonoids exert antioxidant properties. Flavonoids mitigate the risk of heart disease by averting the oxidation of low-density lipoproteins (LDLs), reducing platelet aggregation, and enhancing coronary vasodilation [36]. They play diverse roles in preventing cancer development via deactivation of carcinogens, preventing oxidative damage to DNA, suppressing activity of mutagenic genes, inhibiting the cancer-causing activity of different substances, and boosting the detoxification machinery [37]. Citrus flavonoids can inhibit the activation of phosphodiesterase and kinases by regulating the activity of the proinflammatory cytokine TNF-α, which plays a distinct role in the early stages of inflammation [38]. Additionally, flavonoids showcase a range of other activities, notably antibacterial, anti-inflammatory, and anti-hyperlipidemic effects [39].

### 2.2. Terpenoids and Other Chemically Distinct Plant Constituents

Terpenes and terpenoids are commonly classified according to the number of five-carbon isoprene units incorporated into their carbon skeletons. Monoterpenes contain 10 carbon atoms, sesquiterpenes contain 15, diterpenes contain 20, and triterpenes contain 30. Oxygenated or otherwise modified derivatives are generally described as terpenoids. Accordingly, α-thujene is a monoterpene, whereas α-terpineol is an oxygenated monoterpenoid; neither compound should be classified as a triterpenoid [40,41,42].

Triterpenes are C30 compounds biosynthetically derived principally from squalene or related C30 precursors. Subsequent cyclization and structural modification generate diverse triterpenoid families, including lanostanes, dammaranes, cucurbitanes, lupanes, oleananes, ursanes, friedelanes, hopanes, and triterpenoid saponins [41,42,43]. Ursolic acid is an ursane-type pentacyclic triterpenoid and is therefore chemically distinct from α-thujene and α-terpineol.

Plant materials may also contain chemically unrelated bioactive classes that should not be included under triterpenoids. For example, TBP-II is a non-starch polysaccharide; proteins and peptides are amino-acid-based macromolecules; D-fagomine is an iminosugar; D-chiro-inositol and fagopyritols are cyclitols; N-trans-feruloyltyramine is a phenolic amide; and aurantioobtusin, aloe-emodin, rhein, emodin, chrysophanol, and physcion are anthraquinones. These constituents have reported metabolic, antimicrobial, antioxidant, neuroprotective, or anticancer activities [44,45,46,47,48,49,50,51,52,53], but their biological activity does not alter their chemical classification. They should therefore be discussed as separate phytochemical or macromolecular classes rather than as triterpenoids.

### 2.3. Tannins

*Tannins*, encompassing both hydrolyzable tannins (represented by esters of ellagic acid or gallic acid) and proanthocyanidins (condensed tannins), play a pivotal role in shaping the sensory attributes (color and flavor) of fruits and other food products [54]. Blackberries, blueberries, strawberries, and raspberries contain only a small fraction of hydrolyzable tannins [55]. Ellagic acid has been identified in specific fruits by quantifying its overall concentration through evaluation of its content following acid hydrolysis of the ellagic acid derivatives [56]. Ellagic acid is known for its robust antiviral, anti-inflammatory, and anti-proliferative properties, besides exerting protective effects against cancers affecting the colon, lungs, and throat [57]. Gallic acid, a formidable antioxidant abundant in strawberries, raspberries, and red grapes, surpasses the antioxidant activity of vitamin E or C [58]. The three -OH groups of gallic acid, which render it three times more potent an antioxidant than vitamin E or C, facilitate scavenging of free radicals in diseased cells. Gallic acid has demonstrated intriguing benefits, particularly for skin health and for effectively inhibiting cell proliferation and inducing cell death in prostate cancer cells [59].

### 2.4. Stilbenes

*Stilbenes*, recognized for their low molecular weight, are found in diverse fruits that constitute a significant portion of the human diet. They are composed of two phenyl moieties linked by a two-carbon methylene group. Resveratrol is a major stilbene found in large amounts in fruits, preferably grapes, cranberries, blueberries, and others. Its synthesis is mediated by the enzyme stilbene synthase from its precursor molecules, p-coumaroyl CoA and malonyl-coenzyme A (CoA) [60]. Plum fruit has also been recognized as a source of resveratrol, contributing to its widespread natural occurrence in dietary sources [61]. It is one among the extensively researched naturally occurring polyphenolic stilbenes [62]. Resveratrol, a polyphenolic antioxidant, is synthesized by plants and is attributed to their defense against microbial attacks. Additionally, it is produced as a phytoalexin in response to fungal infection, UV radiation, and stress. With a significant amount of resveratrol in red wine, the scientific community has begun to unravel its potential health benefits. Considerable evidence suggests that incorporating resveratrol into the diet can have beneficial effects on aging and various chronic ailments, particularly cancer, Alzheimer’s disease, and diabetes, among others [63].

## 3. Problems in Utilizing Bioactive Compounds

Despite potent health benefits, effective utilization of plant-based bioactive compounds in food, cosmetic, and pharmaceutical products is largely hampered by several factors, particularly their bioavailability, stability, release, and solubility. These factors are summarized as under:

### 3.1. Bioavailability

Improving the bioavailability of bioactive compounds derived from plants hinges on a multitude of factors, encompassing the molecular and chemical properties of these compounds, their interactions with other substances, solubility, and stability across diverse digestive environments. Consequently, many plant-derived products demonstrate reduced bioavailability and restricted accumulation in desired tissues [64]. In response to these hurdles, carriers have been tactically designed to boost the bioavailability of plant bioactive compounds. The carriers have been developed in such a way that they control the size of the delivery system, modify their surface properties, and regulate the release of the payload at the target site. This enables precise and target-specific delivery of the payload in terms of rate and dosage, thereby ensuring effective targeting [65]. This refinement boasts stability, solubility, and effectiveness that extend the pharmacological effect of the bioactive compounds, while mitigating their possible adverse effect [66]. Various methods have been employed to improve the bioavailability of plant compounds by considering their physicochemical characteristics and chemical composition. Lipid-based systems, notably liposomes, are extensively employed for this purpose. Conversely, non-liposomal methodologies that include polymeric nanoparticles [67], nanoemulsions [68], quantum dots [69], micelles [70], and solid lipid nanoparticles have also been employed to deliver the payload to the target site (Table 1).

### 3.2. Stability

The stability factor represents another significant challenge hindering the integration of plant bioactive compounds into cosmetic, food, and pharmaceutical products, typically in the form of extracts. These are susceptible to degradation via processes such as oxidation, hydrolysis, or enzymatic breakdown during storage and processing across diverse conditions. Numerous variables, such as temperature, pH, oxygen concentrations, and the existence of metal ions, impact the stability of these compounds [118]. A variety of plant ingredients, including zedoary turmeric oil [119] and verbascoside, have been utilized to bolster the stability of these compounds. Moreover, an array of other agents, such as phytosomes, ethosomes, nanoemulsions, liposomes, and nanoparticles, have been employed for stability enhancement purposes [120].

### 3.3. Release

The design of the carrier system should enable the accurate delivery of bioactive compounds to a specified site, either at a controlled rate or triggered by specific environmental factors like pH or temperature [121]. In crafting an effective carrier system, the target-specific release of the payload along with carrier biodegradation often stands as a pivotal concern. The mechanism for releasing bioactive compounds from a carrier entails diffusion through the matrix, metabolite desorption, and matrix decomposition [122]. This triggering mechanism, facilitating the release of bioactive compounds from sustained-release formulations, needs to be so efficient that metabolites are systematically liberated from the carrier at a consistent rate. The strategic release process not only streamlines application processes but also guarantees pharmacokinetics that are predictable and reproducible [123]. Compared with the isolated metabolites, the release of the plant bioactive compounds in a synchronized manner regulates the liberation of multiple metabolites within a defined timeframe while preserving their inter-component proportions. This strategy ensures maintenance of the physicochemical traits of plant metabolites, which can otherwise cause asynchronous and unpredictable release that potentially diminishes their bioactivity [124]. Different systems, including liposomes [125,126], nanoemulsions [127], and polymers [128], have been employed in achieving the controlled release of the plant bioactive compounds.

### 3.4. Solubility

The primary obstacle hindering the inclusion of plant bioactive compounds in food, beverages, cosmetics, and pharmaceutical products is their restricted solubility. For instance, carotenoids demonstrate poor solubility, posing challenges for their incorporation into liquid-based products. Similarly, compounds with low oil solubility have difficulty incorporating into oily products [121]. Recent progress in innovating delivery systems for plant-based compounds and their extracts underscores efforts to surmount the challenges mentioned above. Although nanocarriers can address solubility and related formulation limitations, their overall sustainability also depends on how plant bioactives are sourced, extracted, purified, and processed. Sustainable extraction should therefore be considered an upstream component of nanocarrier design.

### 3.5. Sustainable Extraction and Green Fabrication of Phytonanoparticles

The sustainability of phytonanotechnology should be evaluated at the process level rather than inferred solely from the use of plant extracts. Plant-derived metabolites, including phenolics, flavonoids, terpenoids, alkaloids, and tannins, can act as reducing, complexing, capping, or stabilizing agents in nanoparticle formation. However, the environmental profile of the resulting nanomaterial also depends on the source of the plant biomass, extraction solvent, energy requirements, purification steps, reaction conditions, waste generation, and downstream processing. Therefore, the designation “green” should be applied cautiously and should encompass the complete production chain, from biomass selection and extraction to nanoparticle synthesis, purification, formulation, and end-of-life considerations [129,130]. The 12 principles of green chemistry emphasize waste prevention, atom economy, safer solvents and substances, energy efficiency, renewable feedstocks, catalysis, degradation after use, real-time process monitoring, and accident prevention [129].

For plant-based nanotechnology, several of these principles are particularly relevant. Waste prevention can be addressed by maximizing extraction yield and utilizing different fractions of plant biomass rather than discarding potentially valuable residues. Renewable feedstocks can be promoted through responsible use of cultivated or renewable plant materials and, where appropriate, agricultural and agro-industrial residues. Safer solvents and auxiliaries favour water, ethanol, ethanol–water mixtures, or other appropriately selected alternative solvents over highly hazardous organic solvents. Energy efficiency can be improved through process-intensified extraction techniques that reduce extraction time and temperature. Finally, process monitoring and standardization are essential because the chemical composition of plant extracts can vary according to species, genotype, geographical origin, season, plant part, developmental stage, and extraction conditions [131].

#### 3.5.1. Green Chemistry and Green Extraction Principles

Green chemistry and green extraction are related but not synonymous. Green chemistry provides a broader framework for minimizing the environmental and health impacts of chemical processes, whereas green extraction specifically focuses on obtaining valuable compounds from natural matrices while reducing energy consumption, solvent use, waste generation, and environmental impact. Chemat et al. (2012) proposed six principles of green extraction: (i) use of renewable plant resources, (ii) use of alternative and preferably safer solvents, (iii) reduction in energy consumption through process intensification and energy recovery, (iv) conversion of residues into useful co-products rather than waste, (v) reduction in unit operations, and (vi) production of extracts that preserve bioactivity and contain minimal contaminants [130].

#### 3.5.2. Sustainable Extraction Methods

##### Ultrasound-Assisted Extraction

Ultrasound-assisted extraction (UAE) uses acoustic cavitation to disrupt plant tissues and enhance mass transfer between the plant matrix and extraction solvent. The mechanical effects associated with cavitation can increase solvent penetration and facilitate the release of intracellular compounds. UAE can therefore reduce extraction time and, under optimized conditions, decrease solvent consumption compared with conventional extraction procedures. It has been applied to the recovery of phenolic compounds, flavonoids and other plant-derived bioactives [130,132].

##### Microwave-Assisted Extraction

Microwave-assisted extraction (MAE) uses microwave energy to rapidly heat the extraction system. Heating of the solvent and plant matrix can accelerate cellular disruption and mass transfer, resulting in shorter extraction times and potentially lower solvent requirements. MAE has been widely investigated for recovering phenolic compounds, flavonoids, and other bioactive constituents from plant materials [132].

Supercritical fluid extraction (SFE), particularly using supercritical carbon dioxide (scCO_2_), provides an alternative approach for extracting relatively non-polar compounds. Supercritical CO_2_ has favourable mass-transfer properties and can be recovered and recycled, while avoiding the use of large quantities of conventional organic solvents. It is therefore particularly relevant for lipophilic compounds such as certain terpenoids and other non-polar secondary metabolites.

Deep eutectic solvents (DESs) and natural deep eutectic solvents (NADESs) have attracted considerable interest as alternative extraction media for plant bioactives. DESs are generally formed by combining two or more components that interact to produce a mixture with a melting point substantially lower than those of the individual components. NADESs are composed of naturally occurring metabolites such as organic acids, sugars, amino acids, and related compounds. These systems have been investigated for the extraction of phenolic acids, flavonoids, terpenoids, and other bioactive compounds [133]. Their potential advantages include low volatility and the possibility of tailoring solvent composition to improve extraction selectivity. However, the use of DES/NADES should not automatically be labelled “green.” Their viscosity, preparation requirements, recovery, recyclability, biodegradability, toxicity, and compatibility with downstream nanoparticle synthesis need to be evaluated. Moreover, if a DES remains associated with the final extract or nanomaterial, its biological and toxicological characteristics must be considered before biomedical applications are proposed.

##### Hydroethanolic Extraction

For many phenolic and polar phytochemicals, water and water–ethanol mixtures can provide comparatively simple and safer extraction systems. Ethanol is particularly attractive when food-, pharmaceutical-, or biomedical-compatible processing is required. The selection of water or ethanol–water systems can reduce dependence on hazardous volatile organic solvents and can simplify subsequent processing [130]. Nevertheless, solvent choice should be matched to the target chemical class. A universal extraction solvent does not exist because plant secondary metabolites differ substantially in polarity, stability, volatility, and chemical structure. Therefore, extraction should be designed according to the intended phytochemical fraction and its subsequent application in nanomaterial fabrication.

#### 3.5.3. From Sustainable Extracts to Phytonanoparticle Fabrication

Following sustainable extraction, the resulting phytochemical-rich fraction can be employed as a reducing, complexing, capping, or stabilizing system during nanoparticle synthesis. Phenolics, flavonoids, terpenoids, alkaloids, proteins, polysaccharides, and other constituents may interact with metal ions or nanoparticle surfaces through electron donation, coordination, adsorption, or other physicochemical interactions. Plant extracts have consequently been used to synthesize a range of metallic and metal-oxide nanoparticles [134,135].

## 4. Lipid-Based Delivery Systems

**Figure 2:**
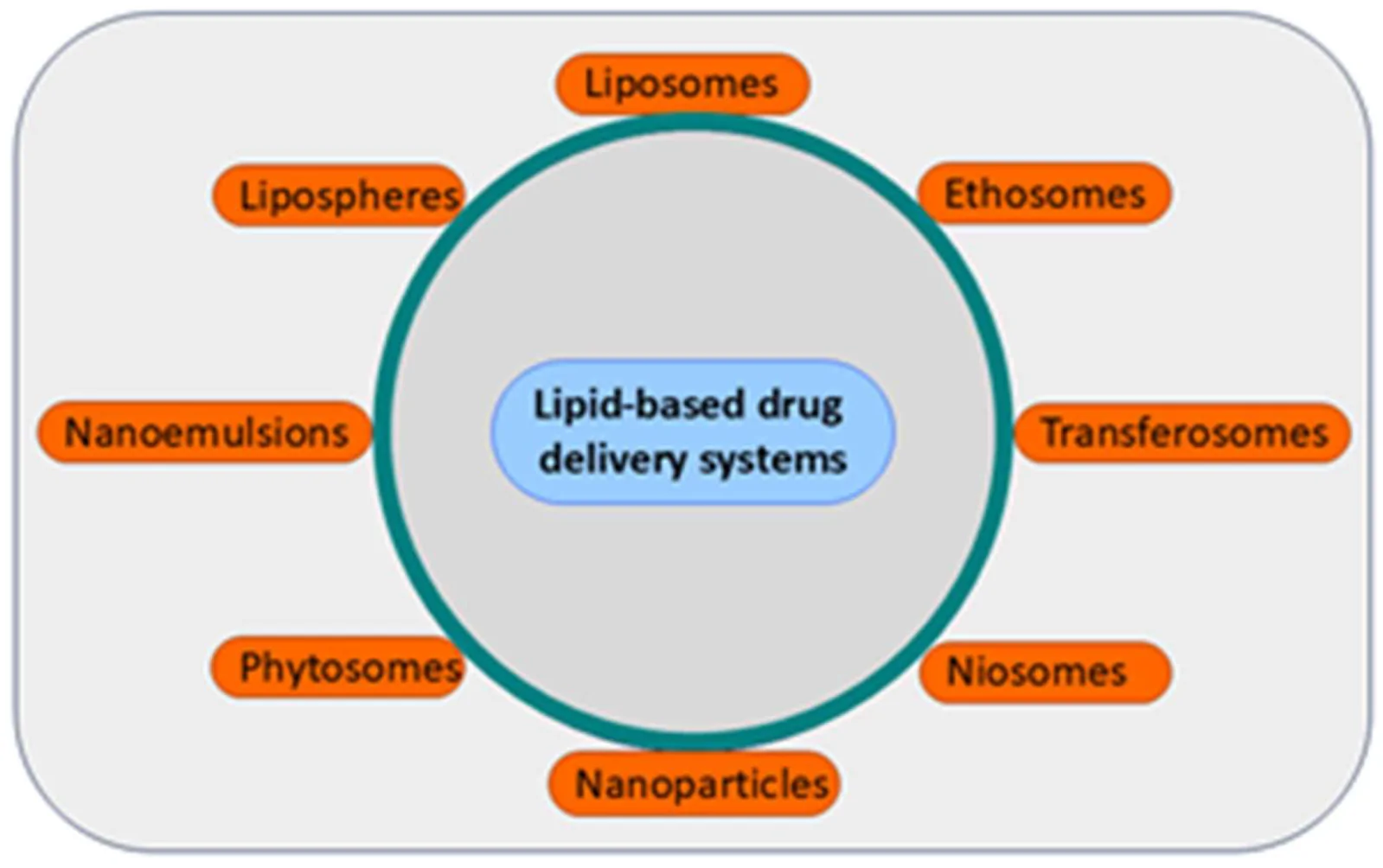
General representation of the lipid-based delivery systems.

Lipid-based nanosystems are widely studied because they can incorporate hydrophilic or lipophilic payloads, protect labile compounds, and permit modification of release and surface properties. Their safety, manufacturability, cost, and performance nevertheless depend on lipid composition, surfactants, particle characteristics, production method, route of administration, and the incorporated bioactives; these attributes should therefore be assessed for each defined formulation [136,137,138,139] (Figure 2).

### 4.1. Vesicular Systems

Vesicular carriers are generated through the assembly of amphiphilic molecules, resulting in an organized system with concentric bilayers [140]. These systems play a crucial role in the transportation and targeted delivery of encapsulated materials. Among the primary and extensively researched categories are liposomes. Ongoing research is dedicated to crafting novel systems with similar functionalities, particularly for plant compounds, offering favorable characteristics. Enclosing these compounds within vesicular structures helps to stabilize, shield, and increase viability within the systemic circulation. This encapsulation approach offers a means to safeguard and prolong the survival of these compounds as they navigate through biological systems [141]. A lot of lipid structures have been formulated and investigated [32].

#### 4.1.1. Liposomes

**Figure 3:**
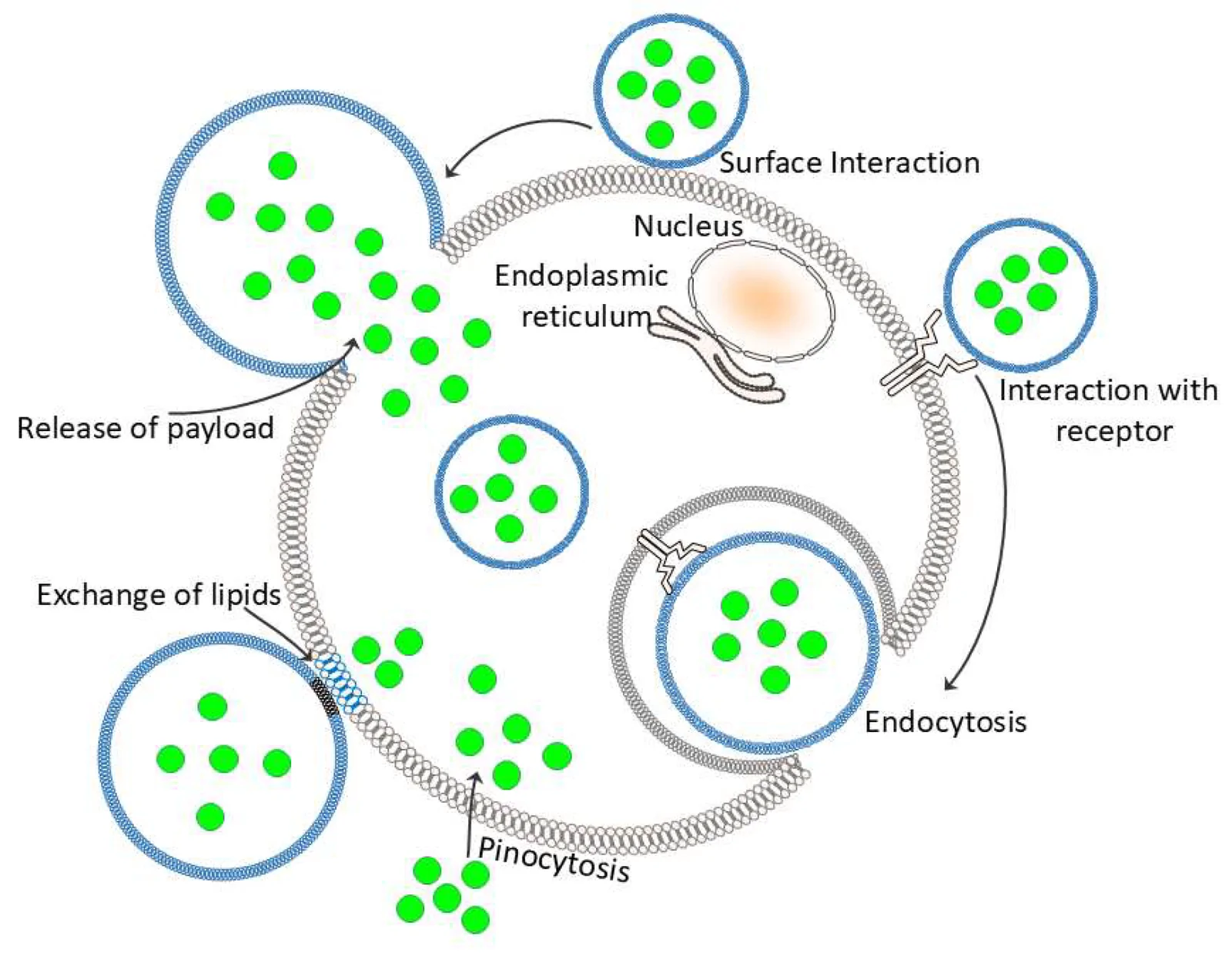
Schematic representation of liposome–cell interactions and therapeutic-payload release.

Liposomes are known for their ease of production and high efficiency, with sizes ranging from nanometers to several micrometers [32]. They are composed of phospholipids or sphingolipids, but may also include sterols like cholesterol and polymers in specific cases. They exhibit versatility in effectively encapsulating and delivering both hydrophilic and lipophilic molecules. The preparation of liposomes is achieved through different techniques, where the temperature is kept higher than the lipid phase transition temperature [142]. Traditional methods, including sonication, hydration, and micro-emulsification, have been extensively employed for liposome production. Nevertheless, newer and more efficient methods have emerged, including the spray drying, freeze drying, and heating methods; osmotic shock method; and the membrane-extrusion method [142]. The vesicular entity of liposomes is broadly classified based on the number of layers and their diameter into unilamellar (single bilayer) and multilamellar vesicles (MLVs; multiple bilayers). Unilamellar vesicles are further classified depending on their size and structure into large unilamellar vesicles (LUVs) and small unilamellar vesicles (SUVs) [143]. As the vesicles typically enclose an aqueous core within a lipid bilayer, they are susceptible to fusion and aggregation that proceeds with premature release of the encapsulated payload following oxidation of the lipids (Figure 3). Additionally, the primary challenges associated with liposomal carriers include their production cost, given that lipidic raw materials tend to be relatively expensive, and issues related to thermodynamic stability. In scenarios involving the encapsulation of multiple molecules, like plant extracts, interactions between the incorporated ingredients within the liposome can significantly influence overall stability and performance [144].

Liposomes have the most extensive clinical experience among the carrier systems; however, clinical use does not eliminate formulation-specific safety concerns. Following systemic administration, liposomes interact with plasma proteins and can undergo opsonization and recognition by cells of the mononuclear phagocyte system, particularly in the liver and spleen. Complement activation is an important consideration for intravenously administered liposomes because it can generate anaphylatoxins such as C3a and C5a and contribute to complement-activation-related pseudoallergy. The response depends on composition, size, surface properties, dose, and recipient factors [145,146,147]. Preclinical assessment should therefore include hemolysis, complement activation, inflammatory responses, biodistribution, and hepatic and renal endpoints. Safety cannot be generalized across liposomes because lipid composition, PEGylation, surface charge, particle size, and drug loading alter protein adsorption, cellular uptake, and immune recognition. Regulatory guidance accordingly recommends product-specific characterization and assessment [148].

**Figure 4:**
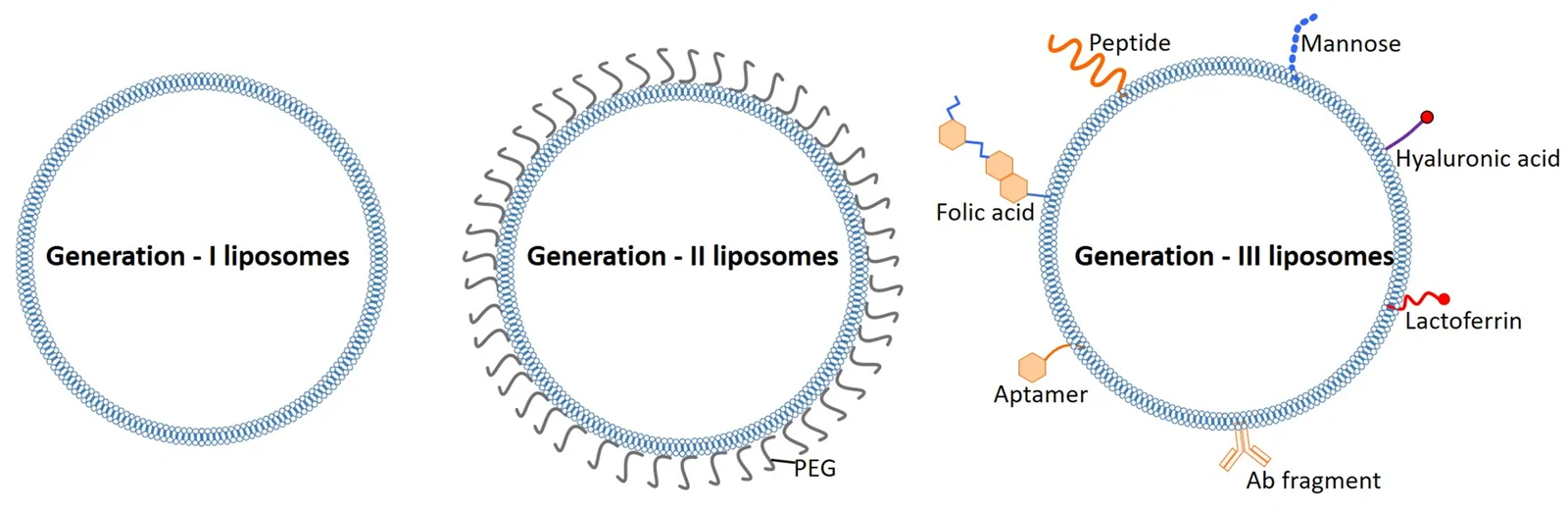
Representation of the composition and development of different liposomes.

As ongoing research in liposomal technology focuses on the development of more specialized encapsulation nanosystems, liposomal carrier systems are categorized into four generations based on their functionalities (Figure 4). First-generation liposomes: These liposomes primarily consist of phospholipids, with occasional incorporation of cholesterol into their structure. Despite challenges such as enhanced uptake by the reticuloendothelial system (RES) and increased susceptibility to chemical degradation [149], they remain a popular choice for delivering plant-based ingredients [150]. *Second-generation liposomes:* It encompasses stealth and stimuli-responsive generation of liposomes. The stealth property of liposomes includes modification achieved with the coating of polymers to adjust their size and charge. An example of this includes polyethylene glycol (PEGylated) coating of the liposomes to enhance their stability and reduce uptake by the reticuloendothelial system (RES), thereby extending the system’s half-life. Stimuli-responsive liposomes can release their contents in response to external triggers, such as changes in pH or temperature. This feature enables them to offer a more targeted and controlled release compared to conventional liposomes. Researchers have successfully used the stealth and pH-sensitive properties of liposomal systems to encapsulate substances such as resveratrol [151] and curcumin [152], thereby providing precise control over their release [153].

*Third generation of liposomes:* It includes specialized systems for incorporating ligands (encapsulated molecules) to enable precise transport to their intended destinations [154]. For example, liposomes loaded with galangin have been precisely engineered to target liver tissue [155]. Similarly, a liposomal system containing curcumin has been formulated with the explicit goal of targeting cancer cells, showcasing the potential of these targeted delivery strategies across various medical applications [156]. *Fourth-generation liposomes*: These liposomes demonstrate multifunctional capabilities, incorporating both diagnostic and therapeutic properties simultaneously, with various strategies utilized for targeted delivery and imaging [157]. Wang et al. [158] developed a specialized system based on magnetic targeting of a liposomal nanocarrier loaded with resveratrol. This carrier system, guided by an external magnetic field, has demonstrated the ability to cross the blood–brain barrier, suggesting potential for treating cerebral diseases. Despite this, there is limited literature on the application of these multifunctional liposomes for plant extracts.

#### 4.1.2. Extracellular Vesicles and Exosomes as Delivery Systems

**Figure 5:**
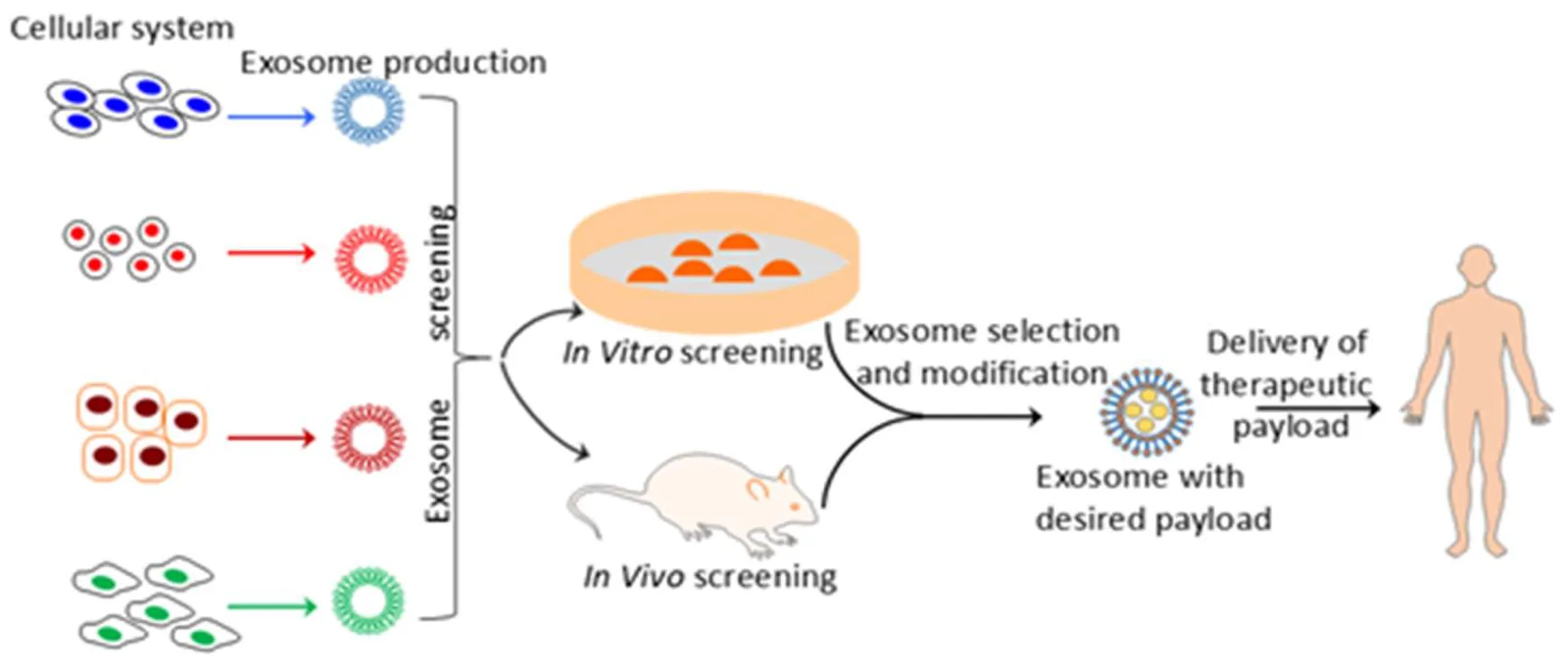
Schematic representation of exosome screening and therapeutic applications.

Exosomes are a subset of extracellular vesicles generated through endosomal pathways and have attracted interest as carriers for nucleic acids and other therapeutic payloads [159]. Their biological origin may provide advantages in cargo protection and cellular uptake, but it does not make them inherently non-immunogenic or exempt them from complement, coagulation, biodistribution, or source-related safety concerns. Composition and biological activity depend on the source cell, culture conditions, isolation and purification methods, associated contaminants, and administered dose. Consequently, any advantage over synthetic carriers must be demonstrated for the defined extracellular-vesicle preparation rather than assumed from its origin [160] (Figure 5).

**Figure 6:**
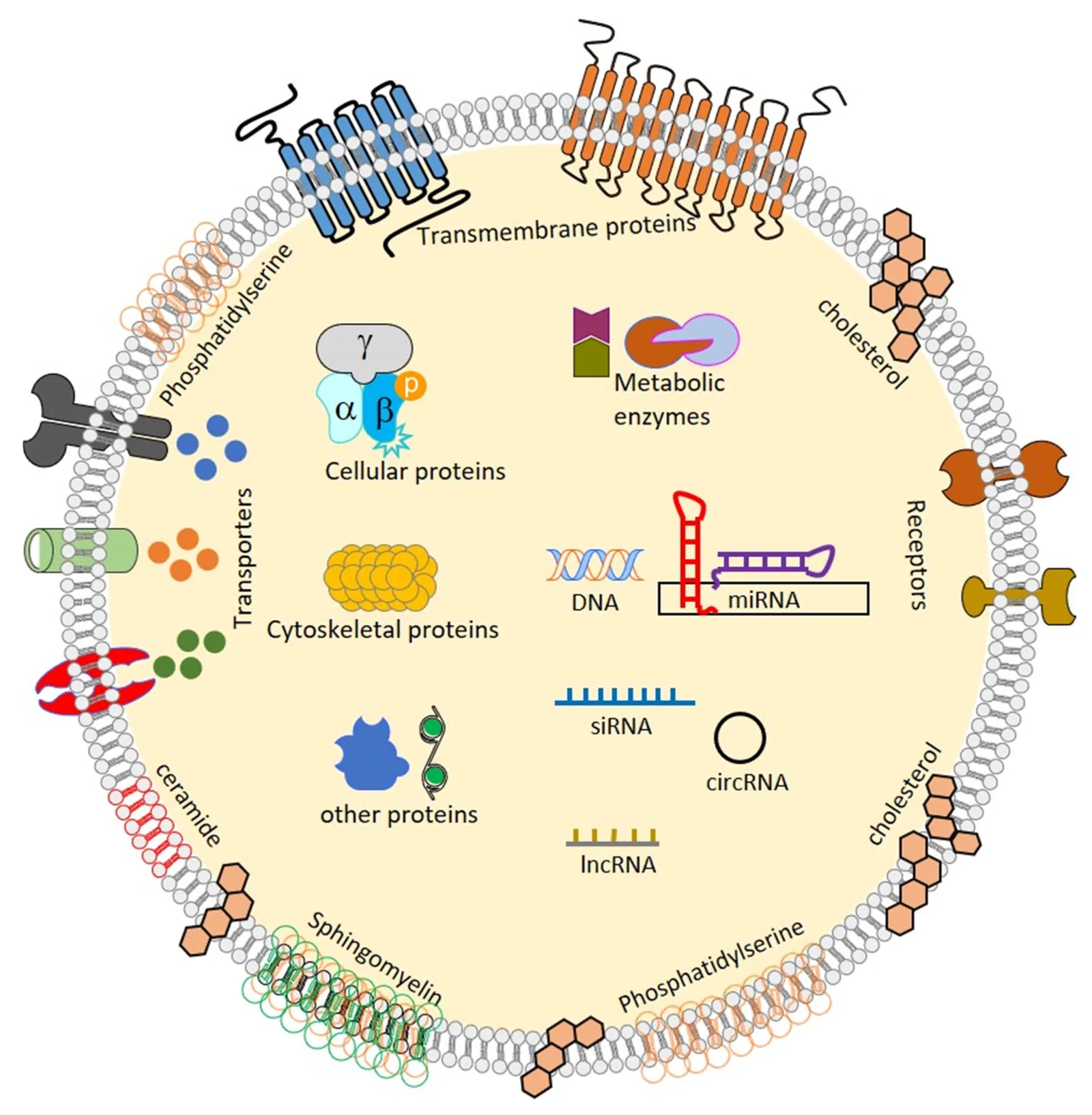
Schematic structure of a biologically derived exosome.

Exosomes loaded with therapeutic payloads require a thorough assessment of the immunological parameters for use as a delivery platform. It was found that HEK293T cell-derived exosomes loaded with nucleic acid miR-199a-3p as a therapeutic entity exhibit a potent anti-invasion and anti-migratory effect, with a slight increase in the cytokine levels against hepatocellular carcinoma [161]. The reduced immunogenicity is ascribed to immature dendritic cells that display lower levels of MHC II molecules, steering T cells for a type 2 T-helper (Th2) and regulatory T-cell response. The ESCRT pathway plays a crucial role in cargo recognition and sorting within exosomes. The intricate process occurs through different complexes: ESCRT-0 for identifying and sequestering ubiquitylated cargo, ESCRT-I and ESCRT-II for inducing membrane deformation that leads to the formation of vesicular buds containing ubiquitylated cargo. The ESCRT-III complex facilitates scission of the vesicle, resulting in the formation of intraluminal vesicles (ILVs). In this whole process, several ESCRT-accessory proteins play a pivotal role in inducing T-cell apoptosis and in promoting a tolerogenic immune response [162]. Exosomes from the tumor cells carrying MHC-I, tetraspanins, LAMP1, and various other immunosuppressive components suppress T-lymphocytes or natural killer cells and facilitate production of the regulatory T-lymphocytes that dampen the immune response [163]. Compared to this, exosomes originating from mesenchymal stem cells lack MHC-I, MHC-II, and other co-stimulatory molecules, preferably CD80 and CD86, and were found to have the ability to evade immune detection. This trait makes them less prone to immune rejection, rendering them more suitable for allogeneic therapeutics [164]. Although several EV preparations have demonstrated low cytotoxicity in defined experimental models, the available evidence does not establish universal safety. Product-specific assessment of cytotoxicity, immunogenicity, thrombogenicity, complement activation, biodistribution, organ accumulation, and repeated-dose toxicity is therefore required (Figure 6).

The natural origin of plant-derived biomaterials, phytochemical carriers, and extracellular vesicles should not be regarded as synonymous with inherent safety or clinical suitability. Although several plant-derived nanocarriers have demonstrated favorable biocompatibility, therapeutic activity, and relatively low cytotoxicity in selected in vitro and animal models, these findings remain context-dependent and cannot be directly extrapolated to humans. The biological response to a nanocarrier may depend on its composition, particle size, surface characteristics, dose, route of administration, purification procedure, associated contaminants, and duration of exposure [165,166]. Plant-derived natural products should not automatically be considered non-toxic simply because they originate from plants. Individual phytochemicals can exhibit dose-dependent toxicity, and their pharmacological effects may change considerably following nanoformulation or prolonged exposure. Nanoparticles containing plant-derived natural products have therefore been investigated not only for their therapeutic potential but also for possible toxicity and adverse biological effects [167]. Consequently, claims of reduced toxicity should be made only when supported by appropriate comparative experimental evidence against a relevant conventional formulation or carrier.

##### Biosafety Considerations

Extracellular vesicles, including exosome preparations, represent biologically active vesicular systems rather than inherently inert or universally non-immunogenic carriers. Their molecular composition can vary according to the source, physiological state, culture or growth conditions, isolation procedure, and purification strategy. Consequently, different EV preparations may exhibit different biodistribution, pharmacokinetic behavior, biological activity, and immune responses [168,169].

Plant-derived extracellular vesicles and exosome-like nanoparticles have been reported to show relatively low immunogenicity in certain experimental settings, but this should be interpreted as a potential advantage rather than an established universal property. Importantly, the current evidence does not justify describing all plant-derived EVs as non-immunogenic or non-toxic. Safety during repeated administration, long-term tissue accumulation, immune recognition, complement activation, and other systemic effects require further investigation using standardized preclinical protocols [170].

##### Batch Variability and Manufacturing Challenges

Another major limitation is batch-to-batch variability. The composition of plant-derived nanocarriers and vesicles can be influenced by plant species or cultivar, geographical origin, cultivation conditions, developmental stage, harvesting time, storage, extraction method, purification procedure, and processing conditions. Consequently, preparations obtained from nominally identical plant sources may differ in particle concentration, size distribution, molecular cargo, purity, and biological activity. These variations present substantial challenges for reproducibility and quality control [168].

For extracellular-vesicle-based systems, heterogeneity and inconsistent isolation procedures remain important obstacles to clinical translation. Conventional isolation methods may produce preparations containing different vesicle populations and non-vesicular contaminants, while differences in purification and characterization procedures can complicate comparisons between studies. Standardized manufacturing, characterization, and critical quality attributes are therefore essential for developing reproducible therapeutic products [165].

##### Scale-Up and Clinical Translation

The transition from laboratory-scale preparation to clinical-grade production represents another major challenge. Scaling up natural nanocarriers while maintaining particle characteristics, purity, cargo composition, potency, sterility, stability, and batch consistency is technically demanding. General nanomedicine research has identified reproducible manufacturing, critical quality attributes, appropriate characterization, batch consistency, and scalable production as important requirements for successful clinical translation [165].

Thus, plant-derived nanocarriers and extracellular vesicles should be described as promising but not inherently safe or clinically mature. Their natural origin may provide potential advantages, including biocompatibility or reduced immunogenicity in specific contexts, but these properties must be demonstrated experimentally for each defined formulation. Future studies should incorporate standardized physicochemical characterization, dose-response analysis, acute and chronic toxicity testing, repeated-dose studies, immunogenicity assessment, biodistribution and pharmacokinetic analysis, reproducible manufacturing, and appropriately designed clinical trials before claims of clinical safety or therapeutic superiority can be substantiated [168].

The spleen, the largest lymphoid organ and a key component of the immune system, is an informative organ for immunotoxicity assessment [171]. In a sustained-dose mouse study, HEK293T-cell-derived extracellular vesicles produced no apparent changes in splenic cell composition [161]. No significant histopathological changes were reported in the thymus, lungs, spleen, heart, liver, kidneys, adrenal glands, ovaries, or uterus, and hematological testing showed minimal differences in erythrocytes, leukocytes, platelets, neutrophils, lymphocytes, monocytes, hemoglobin, and 14 other blood markers between treated and control groups [161]. Exosomal surface molecules can also modulate cytotoxic immune responses [172]. For example, placenta-derived exosomes carry natural killer group 2 member D (NKG2D) ligands that downregulate NKG2D on cytotoxic effector cells [173].

Although some exosome preparations have shown low toxicity in preclinical models, their safety and clearance remain source-, dose-, and formulation-dependent. Exosomes can be cleared rapidly from the circulation by macrophages of the mononuclear phagocyte system, with kinetics comparable in some settings to synthetic liposomes [174]. Rapid macrophage-mediated clearance after intravenous administration has been reported for exosomes derived from bone-marrow dendritic cells [175], splenocytes [176], and rat pancreatic adenocarcinoma cells [177]. Surface engineering, including PEGylation, has therefore been explored to reduce hepatic clearance [178]. Some preparations express surface proteins such as CD47 that may reduce macrophage uptake through signal-regulatory protein interactions; however, this effect is source- and context-dependent [179].

Delivering therapeutics across the blood–brain barrier remains a major challenge in the treatment of neurological disease. Exosomes derived from endothelial bEND.3 cells and dendritic cells have transported small-molecule drugs or siRNA across the blood–brain barrier in preclinical models [180,181]. Characterizing post-administration biodistribution is nevertheless necessary for dose selection, route optimization, and identification of off-target exposure. Source-dependent surface proteins may influence tissue tropism—for example, exosomes from thymic epithelial cells carrying Wnt4 accumulated in the mouse thymus [182]. Hypoxia-targeted tumor-derived exosomes loaded with olaparib also increased apoptosis and inhibited tumor progression in an in vivo model [183]. Surface engineering can further alter targeting, as illustrated by rabies viral glycoprotein-modified exosomes investigated for neurological delivery [184]. Curcumin-primed exosomes reversed lipopolysaccharide-induced pro-inflammatory gene expression in buffalo granulosa cells [185], and curcumin increased exosomal TCF21 in a lung-cancer model [186]. Berry anthocyanidins, including petunidin, peonidin, cyanidin, delphinidin, and malvidin, have likewise been evaluated as exosomal cargo [187].

RNA delivery has been investigated using both synthetic carriers and exosomes. Synthetic carriers can offer high yield and scalable production, whereas their broader use may be constrained by formulation-dependent toxicity, instability, immunogenicity, and limited target specificity. Exosomes participate in intercellular communication, but their targeting and immune interactions remain preparation-dependent; surface engineering with targeting peptides may improve performance [188]. Exosomes from plasma [189], cultured human cells [190], aortic endothelial cells [191], and dendritic cells [192] have transported exogenous siRNA in experimental systems. They can also deliver microRNAs, including Epstein–Barr virus-derived miRNAs, to recipient cells [193]. Rabies viral glycoprotein coupled to lysosome-associated membrane glycoprotein 2b improved delivery of miR-124 to ischemic brain tissue [194], while related RVG-engineered exosomes delivered nerve growth factor mRNA to the ischemic cortex and improved preclinical outcomes [195]. Exosomes carrying three-way-junction RNA nanoparticles have also been evaluated for survivin-targeted RNA delivery [196].

#### 4.1.3. Transfersomes, Ethosomes, Phytosomes, and Niosomes

**Figure 7:**
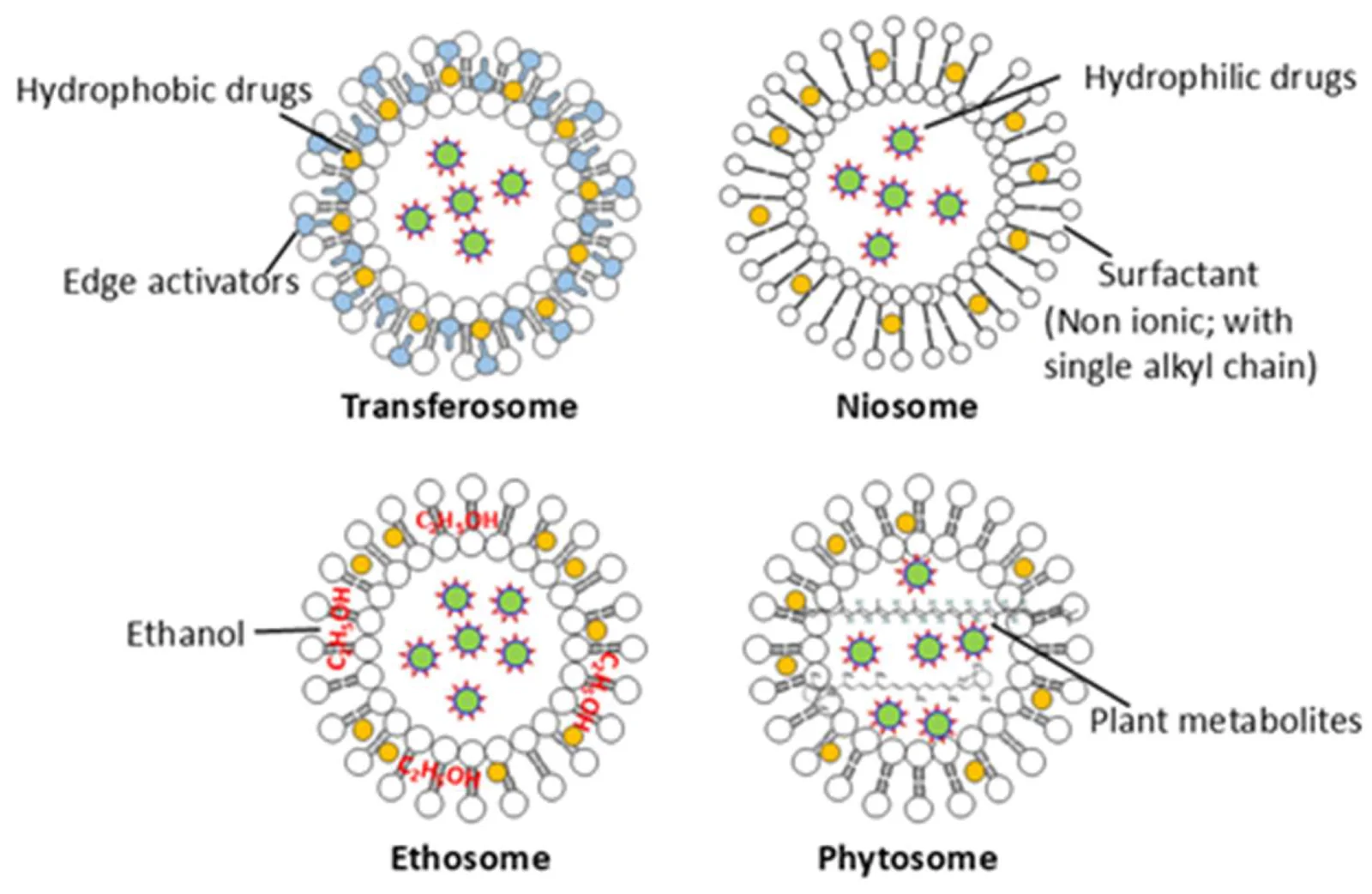
Structural representations of transfersomes, niosomes, ethosomes, and phytosomes.

Advances in vesicular delivery have produced several specialized carrier classes, including transfersomes, ethosomes, phytosomes, and niosomes [197] (Figure 7). Transfersomes are highly deformable vesicles developed primarily for topical and transdermal delivery [198]. Their phospholipid bilayers incorporate edge-activating surfactants that increase membrane flexibility and facilitate passage through the skin barrier [198]. Avadhani et al. [199] developed EGCG- and hyaluronic-acid-loaded nano-transfersomes that increased ex vivo skin permeation and deposition and showed antioxidant and anti-aging activity in an ultraviolet-radiation model. Ramezani et al. [200] formulated minoxidil- and caffeine-loaded transfersomes for alopecia, reporting improved encapsulation, stability, release, and hair-growth measures. Apigenin-loaded transfersomes similarly showed improved stability, permeability, and prolonged release [201].

Ethosomes are ethanol-rich phospholipid vesicles used mainly for topical and transdermal delivery [202]. Their composition increases vesicle flexibility and can improve the incorporation and skin permeation of lipophilic compounds. Ginsenoside Rh1 from *Panax ginseng* showed improved permeation, retention, and deposition in human cadaver skin when formulated in ethosomes [203]. Phytosomes are phospholipid–phytochemical complexes designed to improve the dispersibility, membrane interaction, and bioavailability of selected plant constituents [204]. Curcumin, silymarin, milk-thistle extract, green-tea constituents, and grape-seed constituents have been incorporated into phytosomal formulations [112]. Niosomes generally combine non-ionic surfactants with cholesterol or related membrane-stabilizing lipids [205]. Their properties depend on hydration temperature, surfactant identity and concentration, composition, and preparation method [206]. Niosomal formulations have been reported for *Myrtus communis* extract [207], morusin [208], marigold extract [209], and curcumin [210]. Niosome safety is formulation-dependent: surfactant chemistry, concentration, surface properties, and route of administration can influence hemolysis and cellular toxicity [211,212,213]. Systemic formulations should also be evaluated for complement and immune interactions, hepatic and splenic distribution, renal effects, repeated-dose toxicity, and histopathological changes [214]. Myrtle essential oil has additionally been formulated in niosomes and evaluated for antimicrobial activity [215].

The favorable biocompatibility reported for some phytosomes is associated with their phospholipid composition and selected bioactive compounds; however, it should not be interpreted as evidence of universal safety. Toxicological outcomes depend on the phytoconstituent and dose, phospholipid composition, formulation characteristics, route of administration, and exposure duration. Improved bioavailability may also increase systemic exposure and alter the safety margin [216,217,218].

Compared with liposomes and lipid nanoparticles, systematic long-term toxicological and organ-distribution data remain limited for many phytosomal formulations. Safety claims should therefore be supported by formulation-specific cytotoxicity, hemocompatibility where parenteral administration is intended, acute and repeated-dose animal studies, hepatic and renal biochemical markers, histopathology, and pharmacokinetic and biodistribution studies. Recent reviews continue to identify safety assessment, manufacturing reproducibility, and clinical translation as priorities for phytosomal systems [218].

### 4.2. Non-Vesicular Systems

Non-vesicular lipid nanocarriers are colloidal particles commonly produced by high-pressure homogenization, emulsification, solvent-based methods, or ultrasonication [219]. Soft colloidal nanocarriers have also been engineered as formulations for photosensitive agents [220]. Their physicochemical versatility supports applications in drug delivery, bioimaging, gene transfer, and antimicrobial formulations. Suitability for oral, intravenous, pulmonary, or topical administration remains dependent on composition, particle properties, and formulation-specific safety [219]. The principal non-vesicular lipid carriers are outlined below:

#### 4.2.1. Solid Lipid Nanoparticles (SLNs)

**Figure 8:**
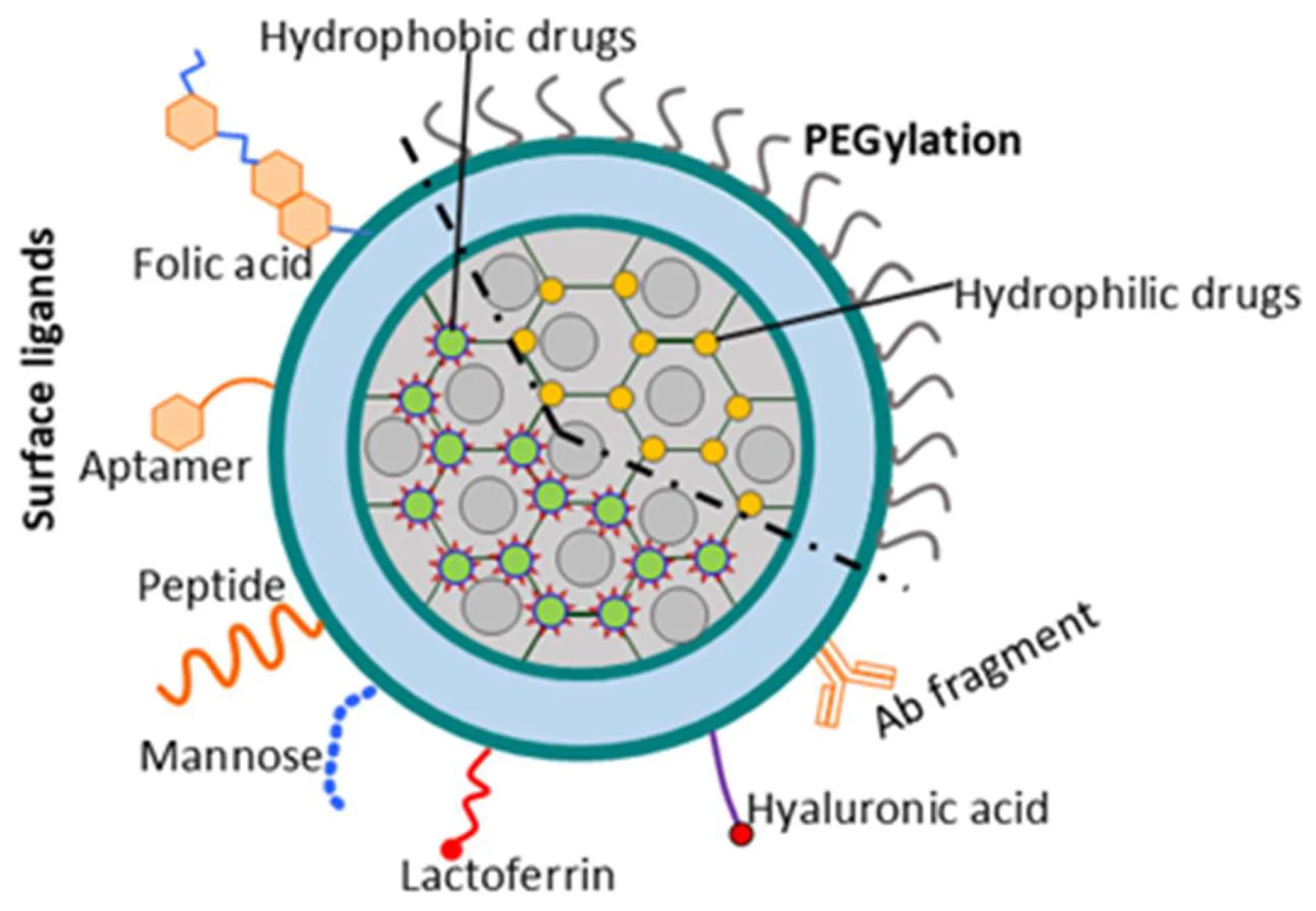
Structural representation of solid lipid nanoparticles (SLNs).

Solid lipid nanoparticles (SLNs) are colloidal carriers in which non-ionic emulsifiers stabilize solid lipids dispersed in an aqueous phase (Figure 8). They are widely studied for payload encapsulation, controlled release, and site-directed delivery [221]. Their biodegradability and apparent biocompatibility depend on the selected lipids, surfactants, particle size, surface properties, dose, and administration route.

SLNs can improve the stability and delivery of plant-derived ingredients, but any reduction in toxicity must be demonstrated comparatively for the defined formulation [221,222,223]. Three distribution models are commonly described: a homogeneous matrix, a drug-enriched shell, and a drug-enriched core [222]. Cationic EGCG-loaded SLNs produced concentration-, time-, and cell-line-dependent antiproliferative effects [224]. Cocoa-butter-based SLNs improved EGCG stability and modified its release [225], while rat studies reported altered pharmacokinetics and bioavailability for EGCG-loaded SLNs [226]. Ban et al. [227] used tristearin and PEGylated emulsifiers to improve curcumin bioaccessibility and oral bioavailability, and Gupta et al. [228] reported improved solubility, stability, permeability, and bioavailability for curcumin-loaded SLNs. Quercetin-loaded SLNs increased gastrointestinal absorption in rats [229]. Resveratrol-loaded SLNs have been evaluated for reducing doxorubicin-associated cardiotoxicity [230], improving controlled release and antioxidant activity [231], and modulating insulin resistance in rats [232]. (+)-Limonene 1,2-epoxide-loaded SLNs have also been assessed for release, antioxidant activity, and cytotoxicity in HaCaT cells [233]. A recent review summarizes the use of SLNs for plant-derived bioactives in cancer-related research [234].

SLNs are often composed of physiologically tolerated lipids; nevertheless, nanoscale formulation can introduce biological effects that are not apparent from the bulk lipid. Relevant endpoints include cytotoxicity, oxidative stress, genotoxicity, and hemolysis. Some formulations show low hemolytic activity, whereas particular lipid or surface compositions can produce substantial erythrocyte damage, emphasizing that safety is formulation-dependent [235,236]. In an in vitro/in vivo study, glyceryl-monostearate SLNs produced cytotoxicity in vitro and were associated with hepatic lipid peroxidation and reduced antioxidant defenses in vivo [237]. Comparative reviews therefore recommend acute, repeated-dose, and long-term toxicity assessment in addition to cell-viability testing [238].

SLNs have also been evaluated for sustained pulmonary delivery of *Yuxingcao* essential oil [239] and for atomized peppermint-essential-oil formulations [240].

#### 4.2.2. Nanostructured Lipid Carriers (NLCs)

**Figure 9:**
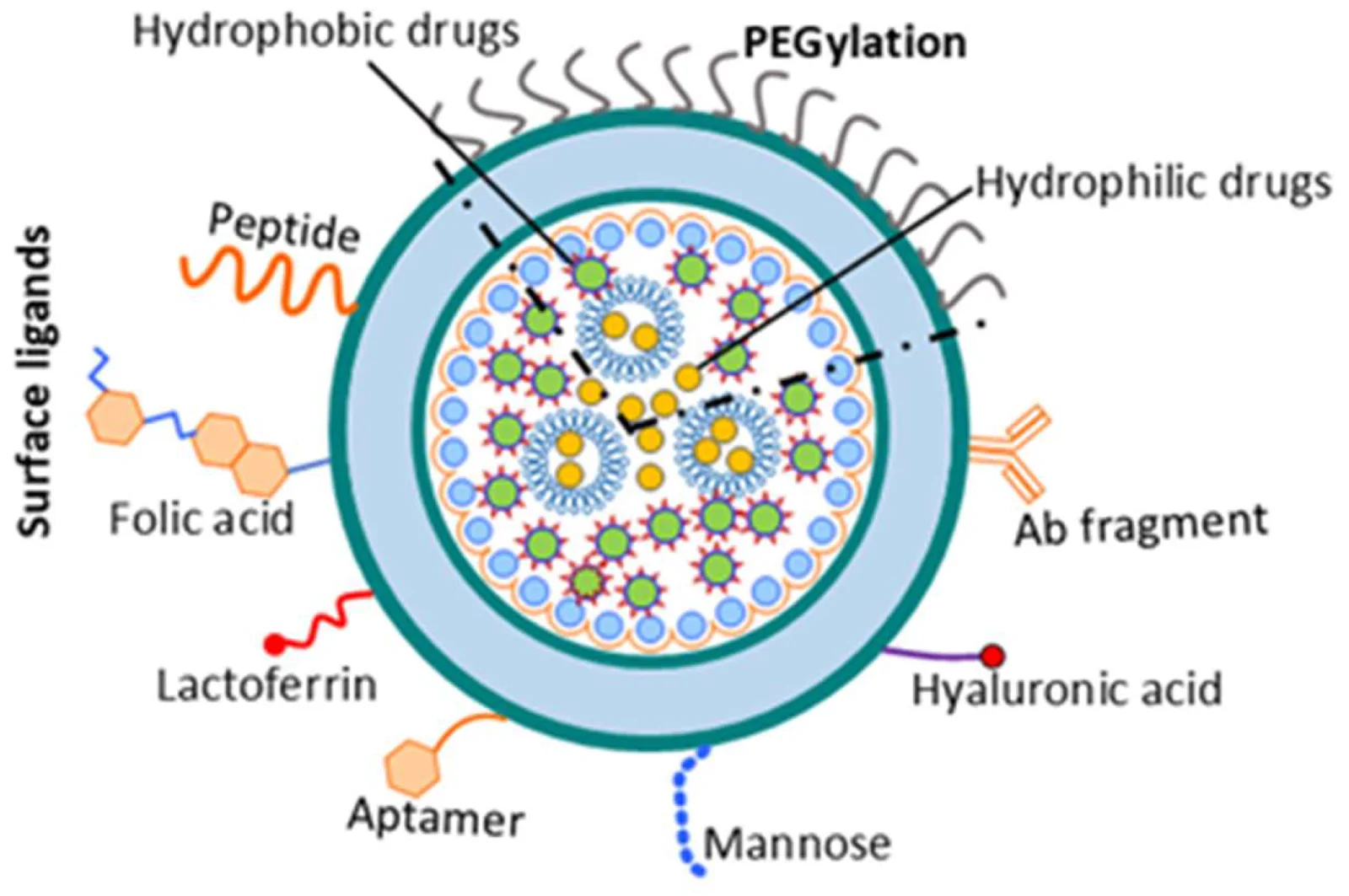
Structural representation of nanostructured lipid carriers (NLCs).

Nanostructured lipid carriers (NLCs) combine solid and liquid lipids to form a less ordered core matrix stabilized by one or more surfactants (Figure 9). The disordered matrix can increase loading and reduce drug expulsion, although performance remains formulation-dependent [241]. Three commonly described NLC structures are the imperfect crystal, multiple, and amorphous models [241]. Silymarin-loaded NLCs have shown improved physicochemical stability and absorption [242], while polyphenol-loaded NLCs have been investigated as anticancer delivery systems [243]. Compared with SLNs, NLCs improved quercetin loading and brain exposure in a preclinical study [244]. Curcumin-loaded NLCs prepared with Peceol and olive oil also enhanced cellular uptake and photodynamic anticancer activity in a breast-cancer cell model [245].

In vitro studies have often reported relatively low cytotoxicity and hemolysis for appropriately formulated NLCs, but contradictory findings occur when cationic surfactants or other reactive components are incorporated. Oxidative stress, inflammatory responses, and membrane interactions are therefore important safety endpoints [246,247]. Following systemic administration, the liver and mononuclear phagocyte system are particularly relevant because nanoparticles can accumulate in hepatic cells and macrophages [248]. Recent NLC studies have accordingly incorporated histopathological, hematological, and biochemical safety endpoints rather than relying only on in vitro cytotoxicity [249].

Additional curcumin lipid formulations have been investigated to improve bioavailability [250], and partially hydrolyzed ginsenoside has been used to modify curcumin-loaded NLCs and regulate in vitro release [251].

## 5. Phyto-Nanomaterials as Carriers

A wide array of phyto-nanomaterials has been found to exhibit pharmacological activities, and converting them into nanoparticles would offer several advantages for biomedical applications. Firstly, given that many phyto-nanomaterials are inherently hydrophobic, their transformation into NPs would significantly enhance their solubility. It could substantially enhance the chances of their administration via intravenous routes for the treatment of a disease. Secondly, NPs derived from phyto-nanomaterials, unlike sub-nanometer parent molecules, possess dimensions in the nanometer range. This characteristic feature would facilitate improved blood circulation and, as such, augmentation in their capability for use in the treatment of a disease [129]. Together, the formulation of nanoparticles (NPs) amplifies the therapeutic efficacy of the original phyto-nanomaterials by enhancing their pharmacokinetic attributes through an increase in interaction with targeted cells and reducing their toxicity to a significant extent. Apart from their pharmacological effects, NPs derived from phyto-nanomaterials exhibit nanostructures suitable for encapsulating beneficial molecules via intermolecular noncovalent interactions, including hydrogen bonding, pi-pi stacking, and hydrophobic interactions, allowing exploitation of phyto-nanomaterial-derived NPs for use as carriers for drug delivery purposes.

Polyphenols are increasingly incorporated into functional polysaccharide films and other delivery materials [252]. These plant metabolites are widely distributed in vegetables, fruits, seeds, tea, and medicinal herbs, and their occurrence and recovery depend on biosynthetic and extraction factors [253]. Beyond their intrinsic pharmacological activity, polyphenols can serve as structural or functional components of biopolymer-based and particle-engineered delivery systems [254,255].

### 5.1. Protein–Polyphenol-Based Nanodrug Delivery Systems

The interaction of polyphenols with proteins has long been exploited in processes such as leather tanning and is now being adapted for delivery and functional-material applications [255,256]. Proteins offer broad availability, diverse functional groups, and potentially high loading capacity. Protein–polyphenol association can improve stability and alter biological performance, but it is governed by the structures and concentrations of both components [257,258,259]. Aromatic and hydrophobic amino-acid residues provide binding sites for polyphenol groups, while polar carbonyl, amino, and hydroxyl groups support hydrogen bonding [257,258,259]. Under alkaline oxidative conditions, quinone intermediates can also form covalent links with nucleophilic amino-acid residues [259,260,261]. EGCG–gelatin layer-by-layer films and microcapsules exemplify this approach and can contain substantial EGCG within the capsule membrane [262]. EGCG has also been combined with protamine for siRNA delivery in drug-resistant breast-cancer models [263]. Amino-acid-induced EGCG nanoparticles prepared with arginine, lysine, glycine, alanine, or glutamic acid showed strong antioxidant activity and inhibited 4T1 tumor growth in preclinical models [264].

Protein therapeutics can lose activity through hydrolysis, denaturation, or intracellular trafficking barriers. Polyphenol-assisted assembly offers one strategy to protect protein cargo and promote intracellular delivery. Tannic-acid-mediated protein nanoparticles have shown serum stability, charge conversion, endosomal escape, and intracellular release through competing supramolecular interactions [265].

PEG–EGCG micellar nanocomplexes carrying trastuzumab (Herceptin) prolonged circulation and enhanced tumor suppression in BT-474 xenograft-bearing mice compared with free protein [266]. Luo et al. [267] used a tannic-acid-coated, genipin-crosslinked human-serum-albumin carrier to protect curcumin during oral administration and target the inflamed colon. Bovine-serum-albumin/tannic-acid multilayer capsules likewise enabled enzyme-responsive release of hydrophobic cargo with low cytotoxicity [268]. Han et al. [269] used tannic acid to assemble several proteins, including pepsin, hemoglobin, and glucose oxidase, and showed that hydrogen bonding, hydrophobic interactions, and ionic interactions contribute to capsule stability. Amyloid fibrils coated with polyphenols can form reversible antibacterial hydrogels [270]. EGCG-loaded amyloid nanofilaments traversed the gastrointestinal tract, accumulated in the colon, alleviated experimental colitis, and modulated barrier function and the gut microbiome in mice [271].

### 5.2. Metal–Polyphenol-Based Nanodrug Delivery System

Polyphenol–metal coordination is important in both biological and materials contexts [272]. The o-hydroxyphenyl and m-hydroxyphenyl groups of polyphenols can act as multidentate ligands and form chelates with metal ions [273,274]. Coordination depends on pH, metal identity, stoichiometry, and polyphenol structure; Fe^3+^, for example, can form mono-, bis-, or tris-ligand complexes as pH increases [273,274]. Removing a sacrificial template coated with a tannic-acid/metal layer produces hollow capsules whose stability and thickness depend on the selected metal [116]. Building-block composition also influences the morphology and mechanical properties of metal–phenolic networks [275]. Catechol binding to TiO2 can generate bidentate bridging or chelating geometries [276]. Metal–phenolic systems have demonstrated antimicrobial and anti-biofilm activity [277] and have been investigated as multifunctional drug-delivery and cancer-theranostic platforms [278]. Polyphenol-assisted assembly has also been used to construct nanoparticles containing platinum prodrugs for cancer therapy [279].

### 5.3. Small Molecule–Polyphenol-Based Nanodrug Delivery Systems

Traditional herbal decoctions contain nanoscale or colloidal assemblies composed of polyphenols and other small molecules [280]. This self-assembly can be exploited in delivery design. Berberine, tannic acid, and glycyrrhizin form organic colloidal particles through noncovalent interactions; isothermal titration calorimetry indicates strong tannic-acid–berberine binding that is further stabilized by glycyrrhizin [281]. Berberine and selected plant-derived small molecules can also form nanofibers or nanoparticles through hydrophobic and electrostatic interactions. These assemblies showed morphology-dependent toxicity and antibacterial activity in zebrafish and bacterial models [282]. Such findings support the potential of carrier-free or low-excipient supramolecular systems, but their composition, stability, pharmacokinetics, and comparative safety require formulation-specific evaluation.

### 5.4. Nucleic Acid–Polyphenol-Based Nanodrug Delivery Systems

Gene therapy and RNA interference can modulate disease-related gene expression, but nucleic-acid instability, high molecular weight, membrane impermeability, and intracellular trafficking limit effective delivery [283]. Reliable delivery systems are therefore required [284]. Viral and non-viral approaches, including lipid and polymeric nanoparticles, have been widely investigated [283,284,285]. Polyphenols can participate in nucleic-acid delivery through reversible hydrophobic, hydrogen-bonding, and electrostatic interactions [114]. In one core–shell system, EGCG complexed with siRNA to form an anionic core that was condensed with a low-molecular-weight cationic polymer; the formulation suppressed inflammatory-gene expression and attenuated chronic intestinal inflammation in a preclinical model [286]. EGCG has also facilitated delivery of single-stranded oligonucleotides by forming anionic cores that are coated with cationic polymers, thereby improving cellular internalization and intracellular delivery [287]. Tannic acid can function as a reversible molecular glue for DNA hydrogels; ester-rich tannic acid contributes degradability, and the resulting material showed hemostatic activity in vivo [288].

### 5.5. Comparative Perspective and Barriers to Clinical Translation

Although the carrier systems discussed above demonstrate substantial potential for improving drug solubility, stability, biodistribution, cellular uptake, and site-specific delivery, their translational potential differs considerably among carrier classes. Lipid-based systems, particularly liposomes and lipid nanoparticles (LNPs), currently have a relatively stronger translational position, supported by their extensive preclinical and clinical development and the availability of clinically established lipid-based products. However, their successful industrial development still requires stringent control of particle size, size distribution, drug-loading/encapsulation efficiency, lipid composition, sterility, storage stability, and other critical quality attributes. Stability, sterilization, regulatory compliance, reproducibility, and quality control remain important challenges for the large-scale production of lipid-based nanoparticles [289,290].

Polymeric nanoparticles, polymeric micelles, and dendrimers provide considerable flexibility in controlling particle size, surface functionality, degradation, and drug release. This versatility can also increase formulation and manufacturing complexity. Compared with lipid-based nanotherapeutics, polymeric nanotherapeutics have undergone more limited clinical translation, and reproducible preparation of consistent, homogeneous batches remains a major barrier. Additional challenges include purification, endotoxin control, long-term storage, physicochemical characterization, and regulatory compliance [291].

Inorganic and hybrid nanocarriers offer structural stability, tunable surface chemistry, and imaging or theranostic functionality, but increasing complexity also creates challenges in physicochemical characterization, long-term safety assessment, control of surface functionalization, and reproducible manufacture. The major translational limitation is often not laboratory-scale efficacy but conversion of a complex formulation into a reproducible pharmaceutical product. Changes in raw materials, composition, mixing, solvent conditions, temperature, or purification can alter particle size, surface properties, loading, release, and biological performance [165,292].

Good Manufacturing Practice (GMP)-compliant production and batch-to-batch reproducibility are critical barriers to clinical translation. Development requires definition of critical quality attributes (CQAs), control of critical process parameters (CPPs), validated analytical and manufacturing methods, and evidence of consistent product quality. Because nanocarrier properties influence pharmacokinetics, biodistribution, safety, and efficacy, comprehensive physicochemical, chemical, and biological characterization is required, and scale-up should be considered from the early stages of formulation development [292]. Representative clinical evidence and the principal limitations of these delivery platforms are summarized in Table 2.

## 6. Design Principles and Therapeutic Applications of Phytonanoparticles

Medical research has shown growing interest in delivery systems for naturally occurring bioactive compounds. Polyphenols are especially versatile because their phenolic hydroxyl and aromatic groups support hydrogen bonding, metal coordination, hydrophobic association, π–π stacking, electrostatic interactions, and covalent coupling. They can therefore function as therapeutic payloads or as structural components of delivery systems [255,256]. Polyphenols can assemble with proteins [257,258,259,260,261,262,263,264,265,266,267,268,269,270,271], metals [272,273,274,275,276,277,278,279], small molecules [280,281,282], and nucleic acids [283,284,285,286,287,288], providing substantial design flexibility. These interactions do not, however, establish biological efficacy or safety, which remain formulation-specific.

Nanoparticles (NPs) derived from phytonanomaterials have been assessed not only as therapeutics but also as drug-delivery carriers for treating diverse diseases, including neurological disorders, cancer, infectious diseases, and diabetes.

The successful incorporation of a phytochemical into a nanoscale carrier should not be judged solely by the achievement of nanoscale particle size or high encapsulation efficiency. The appropriate delivery system depends on the physicochemical properties of the phytochemical, the intended biological target, the route of administration, the desired release profile, and the evidence available for biological efficacy. Accordingly, liposomes, solid lipid nanoparticles (SLNs), nanostructured lipid carriers (NLCs), phytosomes, polymeric nanoparticles, and protein-based systems should be regarded as complementary rather than universally interchangeable platforms.

### 6.1. Comparative Characteristics of Major Delivery Systems

Liposomes are phospholipid-based vesicular systems capable of incorporating hydrophilic compounds in their aqueous core and hydrophobic compounds within the lipid bilayer. Their advantages include compositional versatility and modifiable surface properties. Conventional liposomes may nevertheless undergo phospholipid oxidation or hydrolysis, payload leakage, physical instability, and limited loading of some phytochemicals [298].

Solid lipid nanoparticles (SLNs) employ a lipid matrix that remains solid at physiological and storage temperatures. They can protect poorly water-soluble phytochemicals and support controlled release, but an ordered crystalline matrix can restrict loading and promote drug expulsion during storage [299].

Nanostructured lipid carriers (NLCs) combine solid and liquid lipids to create a less ordered matrix that can increase loading and reduce drug expulsion. Their performance remains dependent on lipid and surfactant composition, preparation method, and the physicochemical properties of the phytochemical [300].

Phytosomes, or phospholipid–phytochemical complexes, differ conceptually from conventional nanoparticles because they involve molecular or supramolecular association of selected phytoconstituents with phospholipids. This can improve dispersibility, membrane affinity, and oral absorption for some compounds, but the term should not be used for all plant-derived nanoparticles and the benefit is formulation-specific [301].

Polymeric nanoparticles can provide controlled release, protection from degradation, and opportunities for surface modification. Biodegradable polymers such as poly(lactic-co-glycolic acid) can be useful, but burst release, residual solvents, processing constraints, and limited loading of some phytochemicals may remain concerns [302].

Protein-based carriers can exploit hydrophobic, electrostatic, and hydrogen-bonding interactions with phytochemicals. Protein conformation, aggregation, enzymatic degradation, source variability, and gastrointestinal conditions can substantially influence their performance [303,304].

### 6.2. Design Principles for Selecting a Delivery System

The physicochemical characteristics of the phytochemical are major determinants of carrier selection. Hydrophobic compounds may benefit from lipid-based systems that provide a nonpolar incorporation domain, whereas water-soluble compounds may be better accommodated in aqueous compartments, hydrophilic domains, or compatible polymeric matrices, depending on the required release behavior [305].

Particle size can influence dissolution, colloidal stability, cellular uptake, tissue penetration, and biodistribution; smaller particles are not automatically superior. Very small particles have high surface energy and may aggregate without appropriate stabilization, while biological effects remain dependent on route and context [306].

### 6.3. Neurological Disorders

The blood–brain barrier restricts the access of many therapeutic agents to the central nervous system [307]. In a preclinical ischemic-stroke model, glyburide-loaded betulinic acid nanoparticles were designed to exploit cannabinoid receptor 1-related targeting and the antioxidant activity of betulinic acid. Intravenous administration improved brain delivery and outcomes relative to free betulinic acid at an equivalent dose, but these findings remain preclinical and require independent validation and clinical evaluation [308].

### 6.4. Cancer

Self-assembled phytonanomaterials have been investigated as therapeutics and drug carriers in preclinical cancer models [309,310,311,312,313,314]. Carrier-free triterpene and sterol systems have been designed for redox-responsive delivery, photochemotherapy, oral administration, and photodynamic therapy. Some formulations also incorporate chemotherapeutic agents or photosensitizers, but antitumor activity remains formulation-specific and cannot yet be generalized to patients.

#### Passive Targeting and the EPR Effect

The enhanced permeability and retention (EPR) effect has traditionally been used to explain passive tumor targeting. Abnormal tumor vasculature and impaired lymphatic drainage can contribute to the retention of macromolecules and some nanocarriers in selected tumors [315].

The EPR effect is neither universal nor guaranteed. Its magnitude varies among tumor types, patients, and regions within a tumor and is influenced by vascular density, perfusion, permeability, tumor size, stromal and extracellular-matrix organization, interstitial pressure, and lymphatic function [316].

This heterogeneity is important for clinical translation because conventional mouse xenografts may overestimate nanoparticle accumulation relative to many human tumors [316]. A large-scale analysis found that only a small fraction of a systemically administered nanoparticle dose typically reaches solid tumors [317]. Nanoparticles can also enter tumors through active endothelial transport, while the active transport and retention framework incorporates endothelial transport, tumor-component interactions, and lymphatic processes [318,319,320]. EPR should therefore be treated as one context-dependent contributor to tumor delivery.

#### Active Targeting

Active targeting uses ligands such as antibodies, peptides, aptamers, proteins, or small molecules to promote recognition of molecular targets and, where appropriate, receptor-mediated uptake [319].

Active targeting does not allow nanoparticles to reach a tumor independently; the carrier must first circulate and gain access to the tumor or its vasculature. It is therefore complementary to the processes governing tumor localization and tissue penetration [320]. Target heterogeneity, receptor accessibility, ligand density, protein-corona formation, particle properties, and extracellular-matrix barriers can all reduce targeting efficiency [319,320].

#### Tumor-Microenvironment-Responsive Targeting

Tumor-microenvironment-responsive systems are designed to release cargo or change properties in response to pH, hypoxia, reactive oxygen species, redox state, or enzyme activity. These strategies may improve spatial control of release, but the magnitude and selectivity of the trigger must be validated in clinically relevant models [321].

#### Integrated Tumor-Targeting Strategies

Passive, active, and tumor-microenvironment-responsive targeting are mechanistically distinct but can be combined. Tumor delivery is a multistep process involving circulation, tumor access, tissue penetration, cellular recognition, and intracellular release. Rational systems may therefore integrate more than one strategy, but each step should be evaluated rather than assuming that EPR or ligand attachment alone will provide tumor-specific delivery [319,320].

### 6.5. Infectious Diseases

Many phytochemicals exhibit antibacterial, antifungal, or antiviral activity in experimental systems [322], but their unformulated effectiveness may be limited by solubility, stability, or exposure. Berberine can co-assemble with baicalin, cinnamic acid, or related phytochemicals to form nanostructures with enhanced antibacterial activity in preclinical models [282,323,324]. In berberine–baicalin assemblies, the hydrophilic glucuronic acid moiety of baicalin is oriented toward the particle surface and may promote bacterial interactions [282]. Berberine–cinnamic-acid assemblies have likewise shown increased activity against multidrug-resistant *Staphylococcus aureus* in experimental models [323].

## 7. Conclusions

Plant-derived bioactive compounds offer substantial structural and pharmacological diversity, but their therapeutic development is frequently constrained by poor solubility, instability, rapid metabolism, limited bioavailability, and insufficient tissue exposure. Nanocarrier selection should therefore be guided by the physicochemical characteristics of the bioactive compound, route of administration, intended biological target, required release profile, and available evidence of safety and efficacy. No carrier class is universally superior. Liposomes, solid lipid nanoparticles, nanostructured lipid carriers, phytosomes, niosomes, polymeric carriers, protein-based systems, extracellular vesicles, and polyphenol-assembled nanostructures provide different advantages and limitations.

The natural origin of a carrier or payload does not establish safety, biocompatibility, non-immunogenicity, or clinical readiness. Most reported improvements in stability, uptake, bioavailability, and therapeutic activity have been demonstrated in vitro or in animal models. Early clinical studies of liposomal curcumin, bioavailable curcumin dispersions, silybin–phospholipid complexes, and plant-derived extracellular-vesicle preparations demonstrate feasibility, but evidence of clinical efficacy remains limited.

Successful translation will require standardized physicochemical characterization, validated critical quality attributes, comparative pharmacokinetic and toxicological assessment, long-term and repeated-dose safety studies, reproducible Good Manufacturing Practice-compliant production, and appropriately powered clinical trials. Future research should prioritize rational platform selection and clinically relevant comparative evidence rather than whether nanoformulation or natural origin alone will produce therapeutic superiority.

## Figures and Tables

**Table 1:** Representative plant-bioactive delivery systems, therapeutic targets, proposed mechanisms, and reported advantages.

Delivery System	Plant-Derived Compounds	Size Range (nm)	Target	Mechanism	Advantages	References
Liposomes	Curcumin, Quercetin, Resveratrol	50–500	Prostate, breast cancer	Phospholipid bilayers encapsulate compounds; fuse with cell membranes for release	Improves solubility; biocompatibility is composition-dependent	[71]
	Naringin/naringenin	140–166 Not reported	Breast cancer (TNBC radiosensitization)	Lipid vesicles enhance solubility, uptake	Radiosensitizing, cytotoxicity vs free flavonoid	[72]
	Mycophenolic acid + Quercetin	157–183	Breast cancer (MCF-7 and SD-rat models)	Dual liposomes, quercetin modulates MPA metabolism	Improved exposure and antitumor activity in preclinical models	[73]
	Paclitaxel liposomes	50–200	Breast cancer	PEGylation prolongs	Improved tolerability vs solvent PTX, maintained efficacy	[74,75]
	Curcumin + Docetaxel liposomes (CUR-DTX-L)	208.5	Breast cancer (MCF-7, MDR model)	Thin-film hydration, co-delivery strategy	66% tumor inhibition; reduced systemic toxicity	[76]
	Carboxymethyl dextran-coated curcumin liposomes	~150	Cervical cancer (HeLa)	Polymer coating prolongs circulation, protects drug	Cytotoxicity, better retention	[77]
Solid Lipid Nanoparticles (SLNs)	Flavonoids, Essential oils, Terpenoids	50–300	General cancer and antioxidant delivery	Lipid matrix entraps drugs, release via lipid degradation	Stable, enhances oral absorption	[78]
	Quercetin-SLN	134 ± 8	Breast cancer (MCF-7)	SLN encapsulation improves solubility and stability	Apoptosis; IC_50_ vs free quercetin	[79]
	Curcumin-SLN	90–120	Colon cancer (HT-29)	Lipid matrix protects drug, sustained release	Oral bioavailability; stronger antiproliferative effect	[80]
	Berberine-SLN	152 ± 12	Lung cancer (A549)	SLNs increase membrane permeability	Cytotoxicity, migration and invasion	[81]
	Bombesin-conjugated EGCG-SLN	163	Breast cancer	SLN protects EGCG from oxidation	Improved tumor control and survival in a mouse model	[82]
Nanostructured Lipid Carriers (NLCs)	Resveratrol, Curcumin	50–200	Melanoma, breast cancer	Mix of solid and liquid lipids allows higher loading & sustained release	Better stability than SLNs	[83,84]
	Quercetin	100–140	Prostate cancer	Encapsulation reduces degradation	Higher cytotoxicity, controlled drug release	[79]
	Silymarin	120–170	Hepatocellular carcinoma	Lipid nanoparticles enable better absorption	Stronger hepatoprotective & anticancer activity	[85]
Phytosomes	Silymarin	100–500	Liver disorders	Complexation with phospholipids enhances membrane permeability	High oral bioavailability	[86]
	Quercetin-Phytosome	145 ± 10	Breast cancer (in vitro)	Phospholipid conjugation solubility	Improved antiproliferative activity	[87]
	EGCG-Phytosome	~150	Skin cancer (UVB models)	Enhanced stability vs free EGCG	Higher antioxidant & anti-inflammatory effect	[88]
	Curcumin-Phytosome	120–150	Colorectal cancer	Clinical phytosome formulation systemic absorption	Improved plasma levels; clinical tolerability	[89]
Niosomes	Curcumin, Berberine, Flavonoids	80–400	Colon, breast cancer	Non-ionic surfactant vesicles deliver compounds by fusing with cell membranes	Biodegradable, stable	[90]
	Quercetin-PEGylated	90–150	Lung cancer	PEGylation prolongs systemic circulation	Improved tumor accumulation; oral bioavailability	[91]
	Resveratrol	100–160	Colon cancer models	Charge modification improves uptake	Enhanced apoptosis via mitochondrial pathway	[92]
	EGCG-Folate	110–170	Breast & ovarian cancer	Folate ligand promotes receptor-mediated targeting	Greater anticancer efficacy vs free EGCG	[93]
	Berberine-Elastic	80–130	Skin cancer & psoriasis	Elastic niosomes skin penetration & retention	Improved dermal delivery; controlled release	[94]
Transferosomes	Curcumin, Quercetin, catechins	70–200	Transdermal delivery/skin cancers	Ultradeformable vesicles penetrate deep skin/tissue via transdermal route	Enhanced penetration & bioavailability	[95]
	Resveratrol	~100	Skin cancer (UVB-induced)	Phospholipid + edge activator improves skin deposition	Reduced oxidative stress, improved stability	[96]
	EGCG	95 ± 8	Transdermal delivery/skin melanoma (B16F10 cells)	Enhanced skin penetration and sustained release	Potent antioxidant and anti-metastatic activity	[97]
	Silymarin	100–140	Hepatocellular carcinoma	Ultradeformable vesicles target liver	Improved hepatoprotective and anticancer effects	[98]
Ethosomes	Resveratrol, Capsaicin, Quercetin	100–400	Skin cancers, inflammatory disorders	Ethanol-rich vesicles with high flexibility penetrate through skin layers	Better skin permeation	[99]
	Rutin	90–130	Skin permeation/UV protection	Ethanol-based flexible vesicles dermal deposition	Improved stability, antioxidant & anti-photoaging	[100]
Exosomes	Curcumin, Resveratrol, Paclitaxel	30–150	Breast, brain cancer	Nano-vesicles from cells naturally transport compounds to target tissues via receptor-mediated uptake	Potential cargo protection and uptake; product-specific safety required	[101]
	Plant lipids, proteins, and microRNAs	30–120	Gut barrier and gut microbiota	Plant-derived vesicles transport bioactive cargo and can modulate intestinal signaling and microbial composition	Oral-delivery potential; composition and effects are source-dependent	[102,103]
	Therapeutic nucleic-acid cargo	Not consistently reported	Pancreatic cancer and engineered cancer-targeting systems	Engineered exosomes facilitate targeted cargo delivery and cellular uptake	Preclinical targeting potential; source- and formulation-specific validation required	[104,105]
	Quercetin	60–110	Alzheimer’s disease mouse model	Exosomal delivery promotes brain exposure and suppresses phosphorylated-tau-associated neurofibrillary pathology	Improved cognition in mice; preclinical evidence	[106]
	miRNA inhibitor and chemotherapeutic co-delivery	Not consistently reported	Colon cancer and drug resistance	Engineered exosomes enable targeted co-delivery of nucleic-acid and drug cargo	Potential reversal of drug resistance in preclinical models	[107,108]
Nanoemulsions	Essential oils, Carotenoids, Polyphenols	20–200	GI cancers	Oil-in-water dispersions increase solubility and intestinal uptake	Rapid absorption, high stability	[109]
Phytosomes	Polyphenols, Alkaloids, Terpenoids	10–100	Various cancers	Plant extracts act as reducing/capping agents for Ag, Au, ZnO nanoparticles, which target tumor/bacterial sites	Eco-friendly, targeted therapy	[110]
	Curcumin	20–40	Breast cancer	Plant-extract mediated synthesis, surface plasmon resonance	Enhanced cytotoxicity, improved stability	[111]
	Plant-derived bioactives (general)	Formulation-dependent	Oral delivery	Phospholipid complexation improves dispersibility and membrane interaction	Potentially improved bioavailability; formulation-specific evidence required	[112]
	EGCG	25–50	Skin carcinoma	EGCG acts as reducing + stabilizing agent	Antioxidant protection, improved bioavailability	[113]
Nucleic Acid–Polyphenol Nanodrugs	EGCG (green tea catechin), Resveratrol	20–200	Breast cancer, leukemia	Polyphenols interact with nucleic acids to form stable complexes, aiding gene delivery	Synergistic gene-drug therapy	[114]
Small Molecule–Polyphenol Nanodrugs	Curcumin, Resveratrol with chemotherapeutics	50–200	Colon, breast cancer	Polyphenols self-assemble with drugs into nanoparticles, co-delivered to tumor sites	Potential synergy; toxicity must be assessed comparatively	[115]
Metal–Polyphenol Nanodrugs	EGCG-Fe^3+^, Tannic acid–Fe^3+^ complexes	50–200	Melanoma, multiple cancers	Coordination between polyphenols & metal ions forms stable delivery capsules	Biodegradable, pH-responsive	[116]
Protein–Polyphenol Complexes	Tannic acid–BSA, Resveratrol–casein	50–200	Breast, liver cancer	Protein carriers bind polyphenols, improving solubility & stability; release at specific sites	Potential biocompatibility; formulation-specific validation required	[117]

**Table 2:** Representative clinical evidence for formulations and delivery platforms discussed in this review.

Platform/ Formulation	Clinical Setting	Study Status and Size	Main Observation and Limitation	Ref.
Intravenous liposomal curcumin	Healthy volunteers; phase I	Completed; *n* = 50	Dose-related exposure was characterized. Transient erythrocyte-shape changes at higher doses indicated a formulation-specific safety signal; the study did not assess efficacy.	[293]
Intravenous liposomal curcumin	Locally advanced or metastatic cancer; phase I	Published; *n* = 32	The maximum tolerated dose was 300 mg/m^2^ over 6 h. Hemolysis or hemoglobin decline limited higher doses, and no objective RECIST response was observed.	[294]
Theracurmin curcumin nanodispersion	Pancreatic or biliary tract cancer; phase I	Published; *n* = 16	Systemic exposure was increased and short-term tolerability was acceptable; clinical efficacy was not established.	[295]
Silybin–phosphatidylcholine phytosome	Advanced prostate cancer; phase I	Published; *n* = 13	A daily dose of 13 g was recommended for further study. Hyperbilirubinemia was common, and no objective prostate-specific-antigen response was observed.	[296]
Oral grape-derived extracellular-vesicle preparation	Chemoradiation-associated oral mucositis in head and neck cancer; phase I	Registry completed; *n* = 60	The registry indicates completion, but peer-reviewed outcome data were not identified; feasibility should not be interpreted as efficacy.	[297]

## Data Availability

No new data were created or analyzed in this study. Data sharing is not applicable to this article.

## Abbreviations

ASBT, Apical sodium-dependent bile transporter; ATR, Active transport and retention; BA, Betulinic acid; CARPA, Complement-activation-related pseudoallergy; CB1, Cannabinoid receptor 1; COX, Cyclooxygenase; CPP, Critical process parameter; CQA, Critical quality attribute; DC, Dendritic cell; DES, Deep eutectic solvent; DSS, Dextran sulfate sodium; EC, Epicatechin; ECG, Epicatechin 3-gallate; ECM, Extracellular matrix; EGC, Epigallocatechin; EGCG, Epigallocatechin 3-gallate; ESCRT, Endosomal sorting complex required for transport; EV, Extracellular vesicle; GMP, Good Manufacturing Practice; H_2_O_2_, Hydrogen peroxide; HER2, Human epidermal growth factor receptor 2; IBD, Inflammatory bowel disease; ILV, Intraluminal vesicle; LDL, Low-density lipoprotein; LOX, Lipoxygenase; LUV, Large unilamellar vesicle; MAE, Microwave-assisted extraction; MHC, Major histocompatibility complex; MLV, Multilamellar vesicle; MPA, Mycophenolic acid; MPS, Mononuclear phagocyte system; NADES, Natural deep eutectic solvent; NGF, Nerve growth factor; NLC, Nanostructured lipid carrier; NP, Nanoparticle; PDGFR, Platelet-derived growth factor receptor; PEG, Polyethylene glycol; PLGA, Poly(lactic-co-glycolic acid); QU-CAP, Quercetin caprylate; RES, Reticuloendothelial system; ROS, Reactive oxygen species; RVG, Rabies viral glycoprotein; SFE, Supercritical fluid extraction; SLN, Solid lipid nanoparticle; SUV, Small unilamellar vesicle; TB, Tartary buckwheat; TME, Tumor microenvironment; TNBC, Triple-negative breast cancer; UAE, Ultrasound-assisted extraction; UC, Ulcerative colitis; WHO, World Health Organization.
